# Computational Evaluation of Multitarget Capabilities of Phenylethanoid Glycosides Against SARS-CoV-2’s 3CL^pro^ and PL^pro^

**DOI:** 10.3390/ph19071126

**Published:** 2026-07-21

**Authors:** Maria Eduarda Alves Esteves, Bruce Veiga Andriolo, Caio Felipe de Araujo Ribas Cheohen, Thamirys Silva da Fonseca, Mariana Freire Campos, Carla Monteiro Leal, Diego Allonso, Gilda Guimarães Leitão, Suzana Guimarães Leitão, Manuela Leal da Silva

**Affiliations:** 1 Programa de Biologia Computacional e Sistemas, Instituto Oswaldo Cruz, Rio de Janeiro 21045-900, Brazil; meduardaae@gmail.com; 2 Programa de Pós-Graduação em Biotecnologia, Instituto Nacional de Metrologia, Qualidade e Tecnologia, Duque de Caxias, Rio de Janeiro 25250-020, Brazil; 3 Programa de Pós-Graduação Multicêntrico em Ciências Fisiológicas, Universidade Federal do Rio de Janeiro, Macaé 27965-045, Brazil; caiocheohen@ufrj.br; 4 Programa de Pós-Graduação em Ciências Farmacêuticas, Faculdade de Farmácia, Universidade Federal do Rio de Janeiro, Rio de Janeiro 21941-902, Brazil; thamirysfonseca@ufrj.br; 5 Departamento de Produtos Naturais e Alimentos, Faculdade de Farmácia, Universidade Federal do Rio de Janeiro, Rio de Janeiro 21941-902, Brazil; ccamposmariana@gmail.com (M.F.C.); carlam.leal@yahoo.com.br (C.M.L.); 6 Departamento de Biotecnologia Farmacêutica, Faculdade de Farmácia, Universidade Federal do Rio de Janeiro, Rio de Janeiro 21941-902, Brazil; diegoallonso@farmacia.ufrj.br; 7 Instituto de Pesquisas de Produtos Naturais, Universidade Federal do Rio de Janeiro, Rio de Janeiro 21941-902, Brazil; ggleitao@ippn.ufrj.br; 8 Laboratório de Fitoquímica e Farmacognosia, Centro de Ciências da Saúde, Faculdade de Farmácia, Universidade Federal do Rio de Janeiro, Bloco A, Sala 10, 2º Andar, Av. Carlos Chagas Filho, 373—Cidade Universitária, Ilha do Fundão, Rio de Janeiro 21941-902, Brazil; 9 Grupo de Pesquisa em Biologia Computacional, Instituto de Biodiversidade e Sustentabilidade NUPEM, Centro de Ciências da Saúde, Universidade Federal do Rio de Janeiro, Av. São José do Barreto, 764, São José do Barreto, Macaé 27965-045, Brazil

**Keywords:** bioinformatics, computational biotechnology, systems biology, diseases, viruses

## Abstract

**Background:** The development of multitarget drugs capable of simultaneously inhibiting SARS-CoV-2 proteases—3CL^pro^ and PL^pro^—may enhance therapeutic efficacy against COVID-19. Given the historical use of Traditional Chinese Medicine (TCM) in the management of respiratory diseases, phenylethanoid glycosides (PGs) represent an attractive and chemically diverse natural product scaffold for the discovery of antiviral agents. **Objectives**: This study aimed to identify promising candidates within this class capable of simultaneously inhibiting both target proteases. **Methods**: The PG structures described in the literature between 1950 and 2020 were gathered and curated to construct a dedicated database, which was subsequently subjected to virtual screening. In silico ADMETox predictions and 2D ligand–protein interaction analyses were then employed to evaluate the identified hit PGs. Ligand stability within the binding sites of the proteases was further assessed using free energy landscape (FEL) and MM/GBSA calculations, while enzymatic inhibition of the commercial PG was evaluated via FRET assays. **Results**: Virtual screening identified 22 PGs with multitarget potential, predominantly sourced from Asia, followed by the Americas and Europe. The hit compound magnoloside I is found in *Magnolia officinalis*, a species widely used in TCM for respiratory conditions and officially prescribed during the COVID-19 pandemic. A second hit, calceolarioside B, inhibited more than 90% of the enzymatic activity of both proteases in the FRET assay. **Conclusions**: Together, these findings highlight phenylethanoid glycosides as promising scaffolds for dual protease inhibition.

## 1. Introduction

SARS-CoV-2 is an infectious virus with a single-stranded RNA genome that is known to be contagious to humans. It is responsible for causing the illness known as coronavirus disease 2019 (COVID-19) [1]. Many different proteins contribute to the virulence of SARS-CoV-2, with the 3-Chymotrypsin-Like protease (3CL^pro^) and the Papain-Like protease (PL^pro^) being of great importance.

During virus multiplication, the 3CL^pro^ cleaves the viral polyprotein at multiple sites to produce several functional proteins. This protease is a highly conserved cysteine hydrolase found in coronaviruses [2]; the SARS-CoV-2 3CL^pro^, like the proteases of other coronavirus strains, is composed of three domains: domain I (residues 8 to 101), domain II (residues 102 to 184), and domain III (C-terminal domain, residues 201 to 306) [3]. The substrate-binding site is shallow, wide, and located close to the protein surface. It is situated in a cleft region between domains I and II and is formed by the catalytic dyad H41 and C145. Unlike other cysteine proteases, 3CL^pro^ interacts with a water molecule, which updates the third catalytic exception reported for the other proteases in this class [4,5].

SARS-CoV-2 PL^pro^ has a similar structure to SARS-CoV PL^pro^, with the protein structure being divided into 4 sub-domains: a Ubiquitin-Like N-terminal (UBL), an α-helix (Thumb), and two β-hairpins (Finger and Palm) [6,7]. In the Finger domain, there is a zinc-binding region (C189, C192, C224, and C226) [6,7] that, when interacting with a divalent zinc ion, results in the extension of the BL2loop (residues 266 to 271) [7,8], exposing the catalytic triad C111, H272, and D286 responsible for the protease’s ubiquitin and deISGylating activities [6,7,8]. Therefore, the stabilization of the BL2loop would fiscally stop the substrate from interacting with the catalytic triad and therefore inhibiting the protease activity.

Given their importance, inhibiting these proteases could be highly beneficial in combating SARS-CoV-2 infection, and both synthetic and natural compounds have been extensively evaluated with that goal [9]. Among them, nirmatrelvir—a 3CL^pro^ inhibitor—is currently commercialized in combination with ritonavir under the name Paxlovid [10] and is used to treat COVID-19 in multiple countries. Amongst natural products, polyphenols are a class of natural compounds with previously reported antiviral activity. Phenylethanoid glycosides (PGs), or phenylpropanoid glycosides, are polyphenols containing phenylethyl alcohol, caffeic acid, and glycosyl moieties, and several of these compounds, such as forsythoside, verbascoside (acteoside), and calceolarioside B, have shown inhibitory activity against SARS-CoV-2 proteases in in silico and in vitro studies [11,12].

Numerous studies have reported the antiviral potential of plant-derived substances against COVID-19, based on drug repurposing, in vitro assays, and traditional medicinal practices. In China, where the use of medicinal plants has a long-standing history, multiple reports have highlighted the promising effects of herbal formulations, such as decoctions, infusions, and capsules, used in Traditional Chinese Medicine (TCM) for COVID-19 treatment [13,14,15]. PGs are largely present in some of these formulations, acting as antioxidants and free radical scavengers, with applications in anticancer, antiviral, antibacterial, and anti-inflammatory treatments [9,16,17]. For example, the Chinese patented medicine Lianhua Qingwen, which was reported as a potent antiviral agent [18], consists of thirteen herbs, including *Forsythia suspensa* and *Lonicera japonica* [19], both of which contain phenylethanoid glycosides in their chemical composition [20]. In 2023, our group published a screening of a dataset composed of 567 PGs, identifying four compounds that interact with key catalytic residues of PL^pro^ and three with those of 3CL^pro^, suggesting potential inhibitory activity. Notably, five of these PGs are found in plants used in TCM, supporting further investigations of this class as potential anti-SARS-CoV-2 agents [21].

As SARS-CoV-2 has multiple viable targets that can help suppress viral activities, it is ideal for molecules to possess multitarget capabilities. Researchers have been studying molecules capable of inhibiting viral replication and evasion of the immune system, such as quercitrin, which was predicted as having affinity to 3CL^pro^ and PL^pro^ in silico, or baicalin, which has been proven to possess multitarget capabilities in vitro against RdRP and 3CL^pro^ [9].

Nicotinamide riboside has been shown to have affinity to SARS-CoV-2 Spike, 3CL^pro^, PL^pro^, and RdRp in silico [22], further demonstrating the importance and global effort in the search for molecules capable of inhibiting multiple viral proteins.

The emergence of SARS-CoV-2 led to a rush in vaccine and novel drug development to help control the disease. However, dependence on the Spike protein to create immunity and the 3CL^pro^ to inhibit the viral activities increases the risk of obsolescence caused by mutations, as observed with Omicron and its sub-variants [23,24], increasing resistance to Paxlovid [25] and Molnupiravir [26]. As such, a novel drug with multitarget capabilities capable of inhibiting both the 3CL^pro^ and the PL^pro^ would have a higher efficiency in combating SARS-CoV-2 as well as lower chance of selecting mutations that could cause obsolescence of the inhibitor. To this end, a comprehensive literature review encompassing all phenylpropanoid glycosides described between 1950 and 2020 was performed to construct a structural database. We then conducted a virtual screening of this PG library to identify potential multitarget inhibitor candidates against both SARS-CoV-2 3CL^pro^ and PL^pro^.

## 2. Results and Discussion

Redocking experiments showed that all three scoring functions were capable of identifying binding poses with RMSD values below 2 Å relative to the crystallographic ligand. Specifically, for 3CL^pro^, the obtained RMSD values were 1.46 Å for AutoDock Vina, 1.99 Å for GOLD (ChemPLP), and 0.67 Å for GOLD (GoldScore) (see Figure S1). For PL^pro^, the calculated RMSD values were 0.95 Å for Autodock Vina, 0.80 Å for GOLD (ChemPLP), and 0.68 Å for GOLD (GoldScore) (see Figure S2).

**Figure 1:**
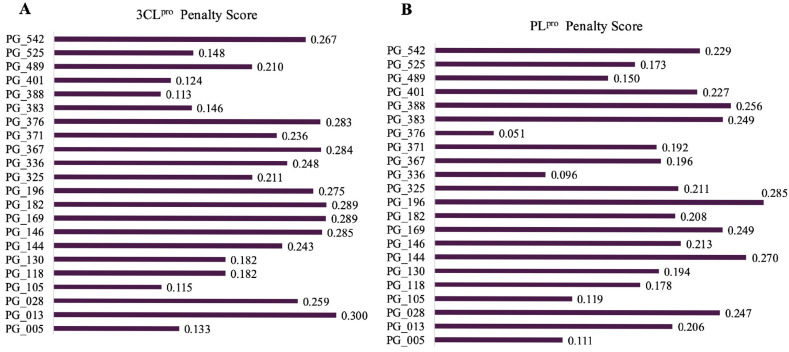
Phenylethanoid glycosides with a penalty score equal to or less than 0.3 with multitarget inhibitory potential for 3CL^pro^ and PL^pro^ of SARS-CoV-2. (**A**) shows the score obtained for 3CL^pro^. (**B**) shows the ranking relative to the PL^pro^ target.

Following this methodological validation, we proceeded with the multitarget screening based on our established compound library. Our previous study [21] demonstrated that phenylethanoid glycosides from TCM may be potential inhibitors of SARS-CoV-2 proteases. Among the 567 compounds tested with 3CL^pro^ and PL^pro^, those predicted with a penalty score of 0.3 or lower were our top candidates, resulting in 22 molecules for both 3CL^pro^ (Figure 1A) and PL^pro^ (Figure 1B), indicating possible multitarget capabilities. These 22 PGs were related to species across 12 distinct plant families, with the Lamiaceae and Oleaceae families showing the greatest representation [16,27,28,29]. Geographically, the distribution of these PGs is predominantly concentrated in Asia, followed by America and Europe, and the least representation in Africa [30]. This distribution strongly reflects the initial database composition, which was heavily influenced by plants commonly used in TCM. The detailed structures of these 22 PGs are provided in Table S1.

### 2.1. In Silico ADMETox Analysis

The molecular mass of the 22 PGs listed ranges from 480.16 Da to 948.29 Da, classifying them as large molecules. According to traditional pharmacokinetic rules [31], a drug must be up to 700 Da to cross cellular barriers. Therefore, it is important to evaluate the standard in which molecules do not fit into such rules. Only Calceolariside B (PG_13) satisfies the MW criterion of 480.16 Da, 11 nHA, 7 nHD, and 10 nRot. The others exceed one or more of these criteria.

Absorption can also be affected when the absorbance is less than 30%, which is the case for all 22 PGs. The Caco-2 criterion also influences absorption, which is shown as all the predicted values were <−5.15 log cm/s. According to the distribution evaluated by Fu, only lippiarubelloside A (PG_05), 6‴-*O*-caffeoyl echinacoside (PG_105), and digiviridifloroside (PG_489) have less than 5% of fractions not bound to serum proteins, making it difficult to efficiently overcome cellular barriers. These findings suggest that physical modifications may enhance the pharmacokinetics of these natural products. The criteria used for selection were based on evaluations of toxicity.

**Figure 2:**
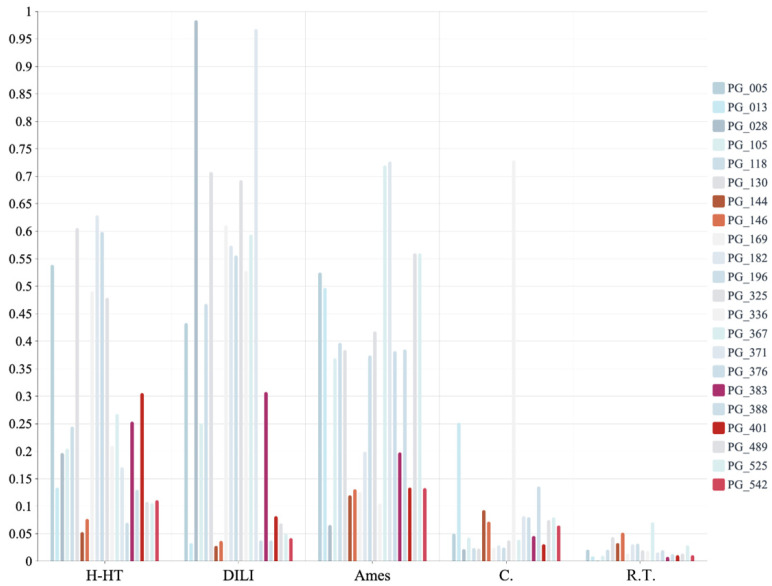
Evaluation of Toxicological Properties. The graph examines toxicity parameters, including human hepatotoxicity (H-HT), drug-induced liver injury (DILI), and results from the Ames test, for mutagenicity, carcinogenicity (C.), and respiratory toxicity (R.T.). A predicted value above 0.3 indicates the potential toxicity of the compound.

From the list of 22 PGs with the lowest penalty score and multitarget activity, we selected and filtered those with the least potential toxicity (see Figure 2). 4-*cis*-*p*-coumaroyl mussatioside (PG_144), 4-feruloyl mussatioside (PG_146), fucatoside C (PG_383), magnoloside I (PG_401), and lagotiside C (PG_542) were found to be non-toxic and non-carcinogenic.

Furthermore, some compounds, such as PG_13, have been found to be toxic. It was detected that PG_13 does not cause liver or respiratory toxicity. However, it does have mutagenic potential, as indicated by the Ames test [32]. This alert should be further investigated in order to enable the detailed identification of the fragment with mutagenic potential. In contrast, 2′,3′,4′,6′-tetra-*O*-galloyl salidroside (PG_028) and 2-(3,4-dihydroxyphenyl)-2-oxoethyl-*O*-α-L-rhamnopyranosyl-(1→6)-(4-*O*-caffeoyl)-β-D-glucopyranoside (PG_371) have a high probability of causing liver injury. However, none of the 22 compounds exhibit respiratory toxicity, which could be extremely fatal for COVID-19 patients, considering the increased fragility of the lungs [33]. Based on the evaluation of toxicity criteria, PG_144, PG_146, PG_383, PG_401, and PG_542 were identified as the most promising inhibitors at this stage of the study. These compounds originate from plants used in TCM or from species endemic to the Americas.

**Figure 3:**
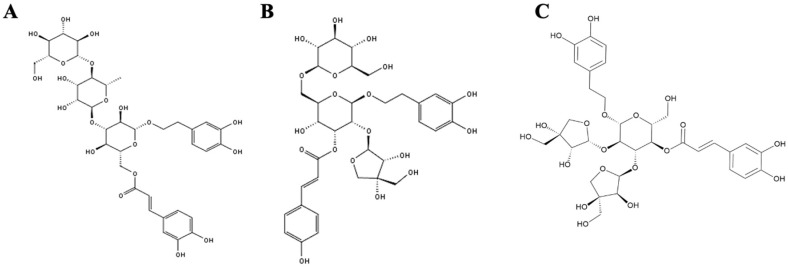
Selected PG structures and sources. (**A**) Lagotiside C (PG_542) from *Lagotis brachystachya* (Scrophulariaceae), used in Tibetan medicine. (**B**) Magnoloside I (PG_401) from *Magnolia officinalis* (Magnoliaceae), employed in TCM for respiratory disorders. (**C**) Fucatoside C (PG_383) from *Lantana fucata* (Verbenaceae), traditionally used in Brazil for colds and inflammation.

PG_542 refers to lagotiside C (see Figure 3A), which is isolated from *Lagotis brachystachya* (Scrophulariaceae family). This species is used as a traditional Tibetan medicine for various conditions, including fever, respiratory and gastrointestinal disorders, and a wide range of inflammatory diseases [34]. Studies concerning lagotiside C have demonstrated significant protective effects on alcohol-injured HepG2 cells [35]; however, its antiviral activities remain largely unexplored.

Magnoloside I (PG_401), illustrated in Figure 3B, is a compound found in *Magnolia officinalis* (Magnoliaceae family), which is endemic to China and employed in TCM for asthma, cough, and other respiratory symptoms [36]. Extensive clinical evidence supports the use of *Magnolia* preparations for asthma treatment, and this plant was officially prescribed during the COVID-19 pandemic in formulas like the Qingfei Paidu Decoction [37,38].

Fucatoside C (PG_383), shown in Figure 3C, occurs in *Lantana fucata* (Verbenaceae family), a species native to the Americas, predominantly Brazil. In Brazilian traditional medicine, the leaves are used as a carminative and anti-inflammatory agent, and also to treat colds and bronchitis. Studies indicate that PG_383 inhibits inducible nitric oxide synthase (iNOS) activity, a crucial anti-inflammatory mechanism [39]. Furthermore, other species within the *Lantana* genus, such as *Lantana camara*, have demonstrated antiviral activity against SARS-CoV-2 [40].

**Figure 4:**
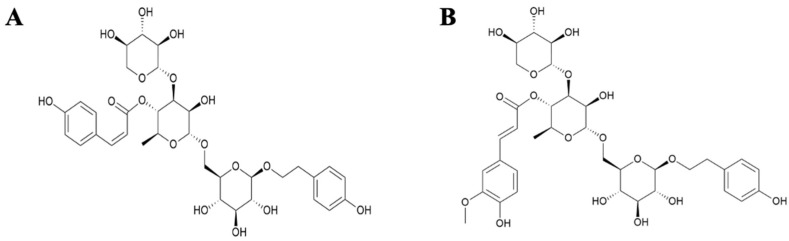
Structures of PGs from *Bignonia* spp. (**A**) PG_144 (4-*cis*-*p*-coumaroyl mussatioside) and (**B**) PG_146 (4-feruloyl mussatioside).

PG_144 (4-*cis*-*p*-coumaroyl mussatioside) and PG_146 (4-feruloyl mussatioside), shown in Figure 4A and Figure 4B, respectively, are compounds isolated from *Bignonia* spp. (formerly genus *Mussatia*, Bignoniaceae family). This genus is distributed across Central and South America, and some parts of North America, being associated with numerous traditional uses. Although some biological activities are documented, the records on the pharmacological activity of both PGs are scarce [41,42].

The pharmacokinetic prediction of the PGs indicates that these compounds may face significant limitations in crossing cellular barriers [31], which compromises their systemic absorption and, consequently, their therapeutic efficacy via the oral route. Although they show limited alignment with classical drug-likeness criteria, these challenges can potentially be addressed through rational structural modification strategies aimed at improving their pharmacokinetic profiles [43]. Nevertheless, the prioritized hit PGs were selected based on their low predicted toxicological penalties and in silico multitarget affinity for both SARS-CoV-2’s 3CL^pro^ and PL ^pro^, making them attractive candidates for further investigation provided their delivery limitations can be overcome.

To translate this therapeutic potential into viable leads, structural optimization can be targeted at specific functional groups. In this context, PGs containing a caffeic acid unit, such as PG_105 (6‴-*O*-caffeoylechinacoside) and PG_401 (magnoloside I), may be optimized through amino acid conjugation [44] with glycine or valine, thereby exploiting intestinal transporters like PepT1 to enhance oral absorption [45]. This strategy reduces reliance on passive diffusion, which is hindered by high molecular weight and high TPSA. In parallel, selective hydroxyl methylation has been demonstrated in simple phenolics [46], like flavonoids, as an effective approach to reduce polarity and improve cellular permeability without compromising antioxidant activity. This second strategy may support the application of this chemical rationale to complex glycosylated phenylpropanoids to overcome pharmacokinetic barriers and broaden their therapeutic feasibility.

### 2.2. 3CL^pro^ Receptor–Ligand Interaction

This section evaluates the affinity and specificity of ligands in relation to the protein. Specifically, we assess the number of interactions between the receptor–ligand complex and the specificity of these interactions. Although hydrogen bonds (H-bonds) are considered weak, they help to stabilize the protein when present in sufficient numbers. Additionally, we aim to detect interactions with specific residues that play a crucial role in enzymatic activity. Structurally, the active site of SARS-CoV-2 3CL^pro^ is divided into four subpockets—S1, S2, S3, and S4—each contributing specifically to substrate accommodation and enzymatic function [47].

As a positive control, we used PF-07321332 (nirmatrelvir), the active component of the antiviral drug Paxlovid, which acts as a SARS-CoV-2 3CL^pro^-specific inhibitor. In this study, the protein–ligand interaction generated with the ChemPLP scoring function was modeled to simulate the covalent bond between the nitrile group of the ligand and the catalytic cysteine of the protease. The docking score obtained was 121.55, and the compound established hydrogen bonds with residues E166, H164, and H41 (details provided in Supplementary Table S2). These interactions highlight the critical role of E166 and H41 in ligand recognition and serve as a benchmark for evaluating the tested phenylethanoids.

Detailed docking scores and the specific interactions between PGs and the target protein residues are provided in Supplementary Tables S2–S7. In particular, the ChemPLP scoring function for PG_144 resulted in a pose that forms H-bonds with Q192 (2.50 Å), T190 (1.85 Å), Q189 (2.63 Å), T24 (2.06 Å), G143 (2.26 Å), N142 (2.37 Å), and L141 (2.03 Å). The GoldScore predicted that the best pose would be the interaction with E166 (1.71 Å and 2.31 Å) and Q189 (2.80 Å). E166 in the S1 pocket plays a crucial role in initiating the enzyme dimerization [48] process by interacting with the N-finger of the opposite chain of 3CL^pro^. AutoDock Vina was the last function tested. This result indicates an interaction with G143 at a distance of 1.98 Å and F140 at 2.38 Å.

The PG_146 compound exhibited significant interactions with key residues located in 3CL^pro^ S1 and S3/S4 active site regions. The ChemPLP pose forms H-bonds with Q192 (2.05 Å and 2.10 Å), E166 (2.07 Å), and Q189 Å (2.04 Å), suggesting a stable positioning of the ligand within critical substrate recognition zones. The GoldScore pose binds with N142 (1.91 Å), T26 (2.61 Å), and Q189 (2.76 Å). Consistently, AutoDock Vina predicted hydrogen bonds with E166 in the S1 subsite (2.11 Å and 1.98 Å), in agreement with the ChemPLP results.

When docked using the ChemPLP scoring function, the third phenylethanoid, PG _383, formed H-bonds with T24 (1.98 Å), T26 (2.10 Å and 2.02 Å), D187 (2.00 Å), F140 (2.11 Å), and Q192 (1.99 Å). The best pose of the same molecule obtained with GoldScore follows the pattern observed with the previously presented PGs and forms a H-bond with E166 (1.92 Å). Additionally, it established a hydrogen bond with C145 (1.92 Å), located in the S1′ subsite between domains I and II, which is part of the catalytic dyad of SARS-CoV-2 3CL^pro^. Finally, the pose predicted by AutoDock Vina revealed hydrogen bonds with T26 (2.04 Å), Q192 (2.36 Å), and E166 (2.53 Å), reinforcing the importance of these residues in the ligand binding.

Calceolarioside B (PG_13) achieved a ChemPLP score of 94.79, forming hydrogen bonds with H164 (2.09 Å), Q192 (1.94 and 2.02 Å), F140 (1.71 Å), and N142 (2.18 Å). The GoldScore model (76.47) predicted interactions with D187 (2.52 Å) and Q192 (2.40 Å), while AutoDock Vina (−8.9) revealed bonds with E166 (1.97 Å) and Q192 (1.83 Å). The recurrent involvement of Q192 reveals its importance as a binding partner, establishing hydrogen bonds with the hydroxyl group located on the lateral phenolic ring of the ligand.

**Figure 5:**
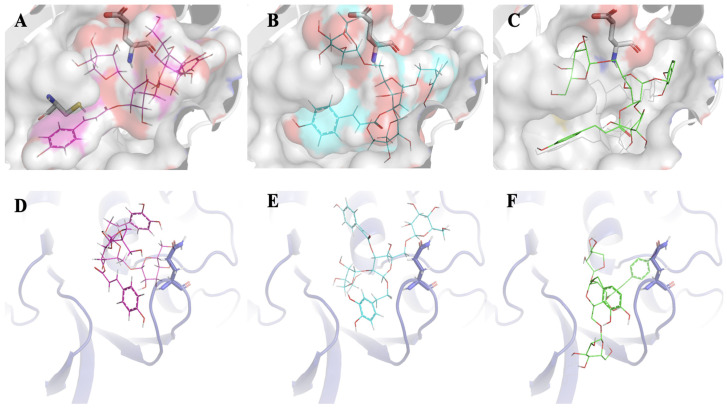
Poses docked for magnoloside I (PG_401) and 3CL^pro^ (**A**–**C**) and PL^pro^ (**D**–**F**) in the three scoring functions. Gray residues in (**A**) are C145 and E166. In (**B**,**C**), E166 is shown, and in (**D**–**F**), Y268 is shown. The line colored in magenta is the PG_401 pose generated by ChemPLP. That in cyan is the PG_401 pose generated by GoldScore. That in green is the PG_401 pose generated by AutoDock Vina.

Among the tested compounds, PG_401 interacts more and forms H-bonds with important residues. The best pose obtained with ChemPLP forms H-bonds with T26, E166, C145 (see Figure 5A), L167, Q192, Q189, and L141. Notably, similar interactions with E166 and Q189 in the S1 and S3/S4 subsites were also reported in the study by Dafydd R. Owen et al. [49], which described the binding mode of the specific protease inhibitor PF-07321332. Consistent with these findings, the best pose obtained with GoldScore also forms H-bonds with E166 (Figure 5B), V186, and M49. Similar to previous functions, the optimal conformation achieved with AutoDock Vina forms H-bonds with E166 at distances of 2.57 Å and 2.17 Å, as shown in Figure 5C, and also with L141 at 2.14 Å. These results reinforce the relevance of E166 as a central residue for ligand binding and suggest that PG_401 may effectively engage both the catalytic and structural regions of the protease.

Finally, the results show that PG_542, as predicted by the ChemPLP scoring function, forms hydrogen bonds with E166 (1.89 Å), G143 (2.80 Å), N142 (2.27 Å and 2.39 Å), T190 (1.70 Å), and N119 (2.13 Å and 2.07 Å), indicating a stable interaction within the active site. GoldScore further supported the relevance of E166, predicting hydrogen bonds with this residue at distances of 2.39 Å and 2.66 Å. Additionally, AutoDock Vina revealed hydrogen bond interactions with Q189 (2.31 Å) and H164 (2.73 Å). While PG_542 demonstrates relevant interactions, PG_401 stands out among the tested phenylethanoids due to its broader engagement with essential residues involved in enzymatic activity and structural stability.

Therefore, in comparison with the positive control, which forms hydrogen bonds with E166, H164, and H41, the phenolic compounds PG_401 and PG_542 demonstrated similar interaction patterns, particularly with E166 and H41. This convergence between the tested compounds and the clinical inhibitor reinforces the relevance of PG_401 as a promising candidate for 3CL^pro^ inhibition.

### 2.3. PL^pro^ Receptor–Ligand Interaction

In our study, we have identified different H-bond interactions between a ligand and amino acid residues. Each score function resulted in interactions with different residues. All of the simulated PGs were found to remain within the BL2loop pocket and interacted with the residues of the region, indicating a possible preference of protein–ligand interaction in that region.

The best pose of the redocked inhibitor GRL0617 generated with ChemPLP had no H-bonds predicted. With GoldScore, it interacted with Y264 (2.44 Å) and Q269 (1.86 Å). Vina pose was predicted to have a H-bond with D164 (2.49 Å) and Q269 (1.77 Å).

The best pose of the phenylethanoid 144 generated with ChemPLP interacted with residues L162 (1.67 Å) and D164 (1.73 Å). With GoldScore, PG_144 interacted with G163 (2.61 Å), G266 (2.27 Å), and Q269 (2.12 Å). Vina pose was predicted to have a H-bond with Q269 (2.12 Å).

For phenylethanoid 146, the pose generated by ChemPLP made H-bonds with residues L157 (2.12 Å), L162 (1.64 Å and 1.87 Å), D164 (1.95 Å and 2.12 Å), E167 (1.88 Å), and Q269 (1.69 Å and 2.12 Å). GoldScore-docked PG_146 made H-bonds with residues E167 (1.61 Å), Q269 (2.18 Å), and D302 (1.53 Å and 2.35 Å). A particularly short non-bonded contact (1.12 Å; typical hydrogen bonds range from 2.5 to 3.5 Å) was also observed with D164, suggesting a strong polar interaction, though covalent bonding was not simulated. PG_146 docked with Vina had no H-bonds predicted.

The pose generated by ChemPLP of PG_383 realized H-bonds with L162 (1.68 Å), D164 (2.14 Å), R166 (2.20 Å), Q269 (1.97 Å), Y273 (2.35 Å), and T301 (1.95 Å). The GoldScore pose interacted with D164 (1.54 Å and 2.35 Å) and Y273 (2.00 Å). Additionally, short-distance non-bonded contacts were observed with Q269 (1.51 Å) and T301 (1.42 Å), indicative of strong electrostatic interactions within the non-covalent docking framework. The interactions predicted with the Vina pose are: R166 (2.31 Å), Y264 (2.47 Å), Q269 (1.96 Å), and Y273 (2.02 Å).

The ligand PG_401 pose docked with ChemPLP (Figure 5D) predicted interactions with L162 (2.06 Å and 2.07 Å), Y264 (2.28 Å), N267 (2.23 Å), Y268 (2.02 Å), and Q269 (1.63 Å, 2.08 Å, and 2.19). This indicates a possible stable interaction with the BL2loop, especially considering the interactions with Y268 and Q269. H-bonds predicted for the PG_401 pose docked with Goldscore (Figure 5E) were made with the following residues: K157 (1.98 Å), E161 (2.23 Å), D164 (1.51 Å), Y268 (1.72 Å), Q269 (1.62 Å), T301 (2.55 Å), and E167 (1.67 Å and 2.68 Å). This indicates a possible stable interaction considering the elevated number of interactions with the BL2loop’s and surrounding residues. The Vina (Figure 5F)-docked PG_401 pose had 4 interactions predicted with residues R166 (2.00 Å), P248 (2.63 Å), Y268 (2.36 Å), and Y273 (2.20 Å).

The ligand 542 pose generated by ChemPLP interacted with K157 (2.23 Å), D164 (2.16 Å), and G266 (2.17 Å). The GoldScore pose predictions included short non-bonded contacts with D164 (1.43 Å) and Y264 (1.48 Å), as well as hydrogen bonds with E167 (1.61 Å), Y264 (1.48 Å), and T301 (2.09 Å). One H-bond was predicted for the pose generated by Vina with L162 (2.42 Å).

These results highlight the significance of the interactions in the binding affinity and specificity between the ligand and the amino acid residue, offering valuable insights into their molecular association. Although all the PGs were predicted to make more H-bonds than the original ligand for PL^pro^, PGs 144, 146, 383, and 542 lacked the ability to make multiple H-bonds with both 3CL^pro^ and PL^pro^, indicating a lower chance of inhibiting the virus proteases. Furthermore, the results also indicate that the molecule PG_401 has the highest chance of possessing multitarget capabilities, with many interactions between the ligand and protein being predicted for 3CL^pro^ and PL^pro^.

### 2.4. Structural Stability of 3CL^pro^–Magnoloside I Complexes

#### 2.4.1. Magnoloside I ChemPLP-Docked

The H-bond analysis revealed persistent interactions between PG_401 and key residues of the 3CL^pro^ active site throughout the three independent molecular dynamics simulations of the ChemPLP-derived complex. During R1, a total of 80 hydrogen bonds were identified, with an average of 4.46 H-bonds along the trajectory. Fourteen simultaneous hydrogen bonds were observed during the first 10 ns of simulation (Figure S3A). Residue Q189 was the most prominent interaction hotspot, exhibiting hydrogen bond occupancies of 45.35%, 27.15%, 2.60%, and 2.45% throughout the simulation. E166 also displayed strong persistence, with occupancies of 36.00%, 1.30%, 8.85%, 1.65%, and 0.05%. In R2, 63 hydrogen bonds were detected, averaging 4.54 interactions. E166 became the dominant interacting residue, forming four H-bonds with occupancies of 9.70%, 72.40%, 12.65%, and 25.40%. The simulation started with approximately eleven hydrogen bonds within the first 10 ns (Figure S3B). Replica 3 (R3) produced 60 hydrogen bonds with an average of 4.27 interactions, reaching a maximum peak of 14 hydrogen bonds shortly after 100 ns (Figure S3C). In this trajectory, E166 again emerged as the most persistent residue, displaying occupancies of 58.05%, 10.60%, 2.83%, and 2.80%. Additionally, H41 contributed significantly through a H-bond with 35.05% occupancy, together with two less frequent interactions showing occupancies of 1.95% and 0.05%. The persistence of interactions involving E166, Q189, and H41 throughout the simulations indicates that PG_401 maintains stable contacts with residues known to play important roles in ligand recognition within the catalytic cavity.

The mobility of PG_401 was investigated through complex RMSD calculations (Figure S4A). Average RMSD values of 4.955 Å, 4.341 Å, and 3.866 Å were observed for R1, R2, and R3, respectively. R1 exhibited the highest conformational fluctuation, with larger oscillations beginning around 110 ns. R2 displayed RMSD values above 5.5 Å shortly after the first 10 ns and reached its largest peak close to 6.5 Å at approximately 170 ns. In contrast, R3 was the most stable trajectory, showing lower average RMSD and smaller fluctuations overall, with maximum peaks of approximately 5.5 Å observed near 50 ns and again around 180 ns. Despite these conformational rearrangements, the ligand remained associated with the active site throughout the simulations.

The structural stability of the catalytic region was evaluated using RMSD and RMSF analyses of residues H41, C145, and E166. Throughout the simulations, R1 exhibited the highest average RMSD among the three replicas (1.678 Å) (see Figure S4B), reaching a maximum value close to 3.0 Å at approximately 120 ns, whereas the lowest value (~0.6 Å) was observed around 80 ns. R2 showed a similar average RMSD (1.677 Å) but with a more uniform behavior and fewer pronounced peaks. The highest RMSD value in R2 reached approximately 2.25 Å at around 150 ns. R3 displayed the greatest structural stability, with an average RMSD of 1.506 Å and a maximum peak of approximately 2.0 Å shortly after 10 ns of simulation. These results indicate that the catalytic machinery remained structurally conserved throughout the trajectories.

The RMSF profiles of the catalytic residues further confirmed the stability of the active site (Figure S4C). Residue H41 exhibited fluctuations of approximately 0.75 Å, 0.70 Å, and 0.60 Å in R1, R2, and R3, respectively. Residue C145 remained highly stable in all simulations, with RMSF values consistently below 0.50 Å. In contrast, E166 displayed greater flexibility, reaching approximately 1.1 Å in R1 and around 0.6 Å in both R2 and R3. Despite this mobility, E166 maintained persistent hydrogen bond interactions with PG_401, highlighting its relevance for ligand stabilization.

The complex PCA revealed distinct conformational behaviors among the three ChemPLP replicas. R1 (Figure S5A) showed an organized conformational transition, with PC1 accounting for 25.6% of the variance and a clear temporal shift from positive PC1 values during the first 50 ns to negative values between 150 and 200 ns. Three main conformational populations were identified—an initial state (PC1 ≈ 10–20), an intermediate state (PC1 ≈ 0), and a final state (PC1 ≈ −25)—indicating a gradual transition toward an energetically favorable bound conformation. In contrast, R2 (Figure S5B) displayed a conformational heterogeneity, with PC1 and PC2 explaining 15.9% and 12.2% of the variance, respectively. R3 (Figure S5C) exhibited an intermediate profile between R1 and R2, with PC1 and PC2 accounting for 10.6% and 8.2% of the variance, respectively. The trajectory gradually shifted from negative to positive PC1 values over time, occupying partially overlapping conformational populations connected by smooth transitions. Although R3 explored a broader conformational space than the final state observed in R1, it remained more restricted than R2, indicating moderate flexibility while preserving a stable bound state throughout the simulation.

**Figure 6:**
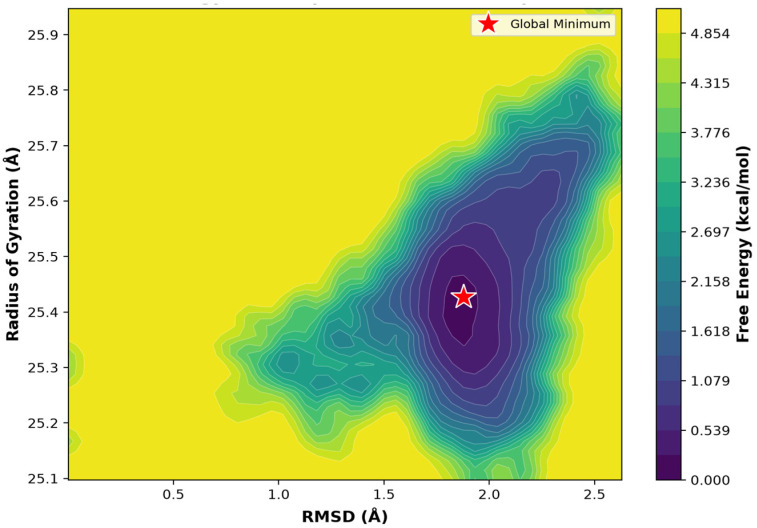
Combined FEL of the ChemPLP-derived 3CL^pro^–PG_401 complex generated from the three independent 200 ns molecular dynamics simulations. The landscape was constructed using the RMSD and Rg as reaction coordinates. The red star indicates the global free-energy minimum, corresponding to the most populated and energetically favorable conformational state.

The combined FEL generated from the three independent ChemPLP simulations (Figure 6) revealed a dominant low-energy region centered at RMSD values of approximately 1.8–2.0 Å and radius of gyration (Rg) values around 25.4 Å, indicating convergence of the trajectories toward similar energetically favorable conformational states. The landscape is characterized by a broad and continuous energy basin extending from lower RMSD values (~1.0–1.5 Å) to the global minimum region, suggesting the existence of interconnected conformational substates separated by low energy barriers. The location of the global minimum within this basin indicates that the most populated conformations correspond to structurally compact states with moderate backbone deviations from the reference structure. The absence of isolated low-energy regions demonstrates that the conformational transitions sampled during the simulations occurred within a common energetic framework rather than through large-scale structural rearrangements.

Binding free energies calculated using the MM/GBSA approach further corroborated the structural analyses. The average binding free energies were −26.89 ± 4.69 kcal/mol for R1, −27.42 ± 4.39 kcal/mol for R2, and −28.46 ± 3.62 kcal/mol for R3, yielding an average binding free energy of approximately −27.59 kcal/mol. The relatively small differences among replicas demonstrate good energetic reproducibility of the ChemPLP-derived binding mode. R3 displayed the most favorable binding energy, whereas R1 showed the least favorable value. Nevertheless, all trajectories remained within a narrow energetic range, indicating that the different conformational substates sampled during the simulations preserve favorable protein–ligand interactions.

Overall, the three independent molecular dynamics simulations consistently demonstrated that the ChemPLP binding pose is structurally and energetically stable. Although differences in conformational flexibility were observed among the replicas, all trajectories maintained persistent interactions with key residues such as E166, Q189, and H41, preserved the integrity of the catalytic site, remained confined to favorable free-energy basins, and exhibited consistently negative MM/GBSA binding energies.

#### 2.4.2. Magnoloside I Gold Score-Docked

The hydrogen bond analysis revealed persistent interactions between the ligand and key residues of the 3CL^pro^-binding site throughout the simulations. The GoldScore-derived R1 complex formed a total of 82 hydrogen bonds, with the highest peak corresponding to 14 simultaneous hydrogen bonds occurring at approximately 100 ns (Figure S3D) and an average of 4.43 hydrogen bonds along the trajectory. Residue E166 showed the highest persistence, displaying five hydrogen bonds with occupancies of 41.35%, 4.20%, 25.20%, 20.45%, and 0.15%, respectively. In R2, 83 hydrogen bonds were identified, again highlighting E166 as the main interaction hotspot. Occupancies of 38.20%, 3.80%, 18.60%, and 1.95% were observed for E166, while H41 also contributed significantly, exhibiting occupancies of 0.10%, 40.30%, and 1.35%. The highest number of hydrogen bonds in R2 was observed near 140 ns, reaching 14 simultaneous interactions (Figure S3E). R3 exhibited a slightly lower total number of H-bonds (75), although the maximum peak reached 15 hydrogen bonds shortly after 80 ns (Figure S3F). Once again, E166 emerged as the most persistent H-bond donor/acceptor, presenting occupancies of 46.05%, 8.15%, and 5.25% during the 200 ns simulation.

The catalytic residues remained structurally stable during all simulations. Their RMSD values ranged from approximately 0.5 to 2.5 Å, with average RMSD values of 1.802 Å for R1, 1.665 Å for R2, and 1.286 Å for R3. Maximum RMSD values of approximately 2.4 Å were observed at 40 ns in R1 and around 50 ns in R2, whereas R3 reached a maximum RMSD close to 2.3 Å near 110 ns (Figure S6A). The RMSF of residues 41, 145, and 166 (see Figure S6B) in the three replicates showed similar fluctuations, with H41 having an RMSF value close to 0.6 Å and C145 varying around 0.5 Å in R1, R2, and R3. Additionally, E166 presented an RMSF of 0.6 Å in R1 and 0.75 Å in R2 and R3. These results indicate that the catalytic machinery remained conserved throughout the simulations.

The complex RMSD analysis demonstrated conformational mobility in all replicates. The average RMSD values were 4.43 Å, 4.45 Å, and 5.23 Å for R1, R2, and R3, respectively. R1 exhibited RMSD values exceeding 7 Å around 60 ns, whereas R2 reached approximately 9 Å near 70 ns and R3 exceeded 7 Å shortly after 60 ns (Figure S6C). Despite this mobility, no evidence of ligand dissociation was observed.

The R1 PCA (Figure S7A) was characterized by two major conformational ensembles separated along PC1 (19.6% of the variance). Early simulation frames (0–50 ns) were concentrated at positive PC1 values, whereas later frames progressively shifted toward negative PC1 values, indicating a clear conformational transition between two dominant bound states through intermediate conformations. R2 exhibited the largest conformational space exploration, with PC1 and PC2 (Figure S7B) accounting for 23.8% and 13.4% of the variance, respectively. The trajectory followed a well-defined temporal progression through three major conformational populations, transitioning from a cluster at high positive PC1 values during the initial stages to intermediate states at lower PC1 and negative PC2 values, and finally to a third population characterized by negative PC1 and positive PC2 values. This ordered pattern indicates extensive but structured sampling of multiple interconnected bound substates. R3 (Figure S7C) showed an intermediate behavior, with PC1 accounting for 24.5% of the variance. The trajectory evolved from positive PC1 values during the early stages toward a broader conformational ensemble located at negative PC1 values at later times. Although multiple conformational populations were observed, they remained connected through continuous transitions, suggesting moderate conformational flexibility while preserving a stable bound state.

The combined FEL (Figure S7D) revealed a broad low-energy basin centered around RMSD values of approximately 1.8–2.2 Å and Rg values between 25.3 and 25.6 Å. The global minimum was located near RMSD ≈ 1.9 Å and Rg ≈ 25.4 Å, indicating that the most populated conformations correspond to compact and energetically favorable protein–ligand complexes. The continuity of the low-energy region suggests that all three replicas converged toward a similar conformational ensemble, supporting the reproducibility and stability of the GoldScore-derived binding mode throughout the simulations.

Binding free energies calculated using the MM/GBSA approach corroborated the structural analyses. The average binding free energies were −28.54 ± 6.31 kcal/mol for R1, −29.62 ± 5.73 kcal/mol for R2, and −26.34 ± 5.17 kcal/mol for R3. Despite the larger conformational variability observed in R2, this replica exhibited the most favorable binding energy, indicating that increased flexibility did not impair ligand affinity.

#### 2.4.3. Magnoloside I AutoDock Vina-Docked

The H-bond analysis revealed persistent interactions between PG_401 and key residues within the 3CL^pro^ active site throughout the three independent MD simulations. Over 200 ns, the AutoDock Vina-derived R1 complex formed a total of 89 hydrogen bonds, with the first major peak occurring within the first 5 ns of the simulation (Figure S3G). Residue E166 was the most prominent interaction hotspot, displaying one highly persistent hydrogen bond with an occupancy of 116.55%, in addition to three other interactions with occupancies of 7.85%, 4.55%, and 0.85%. Residue H41 also contributed significantly to ligand stabilization, exhibiting hydrogen bond occupancies of 40.75%, 8.95%, and 0.25%. Among the three replicas, R2 showed the highest number of predicted hydrogen bonds, totaling 104 interactions. The maximum peak was observed at approximately 70 ns, when 14 simultaneous hydrogen bonds were detected (Figure S3H). Despite the larger number of interactions, individual hydrogen bond occupancies were more distributed. E166 again played a central role, forming four hydrogen bonds with occupancies of 9.05%, 1.25%, 29.75%, and 51.30%. In R3, a total of 92 hydrogen bonds were identified, with the largest peak occurring around 30 ns of simulation (Figure S3I). As observed in the other replicas, E166 remained the dominant interacting residue, forming hydrogen bonds with occupancies of 9.35%, 1.60%, 5.80%, 38.75%, 67.20%, and 0.05%. Collectively, these results highlight E166 as the principal anchoring residue responsible for maintaining ligand recognition throughout the simulations.

The structural stability of key catalytic-site residues was evaluated through RMSD and RMSF analyses. The RMSD values for H41, C145, and E166 exhibited greater fluctuations in R2 than in R1 and R3, reflecting the increased flexibility of this trajectory. The average RMSD values of the catalytic residues were 1.881 Å, 2.145 Å, and 1.693 Å for R1, R2, and R3, respectively. The highest RMSD peak was observed in R2 after approximately 100 ns, reaching nearly 4 Å (Figure S8A). Despite these fluctuations, the catalytic residues remained structurally conserved throughout the simulations. Consistently, RMSF analyses showed similar flexibility profiles among the three replicas. Residue H41 exhibited RMSF values close to 0.6 Å, C145 fluctuated around 0.5 Å, and E166 displayed RMSF values near 1.0 Å (Figure S8B).

Complex RMSD (Figure S8C) analysis revealed pronounced conformational mobility, particularly in R2. The average ligand RMSD values were 3.567 Å, 7.270 Å, and 3.810 Å for R1, R2, and R3, respectively. R1 reached approximately 5 Å at 180 ns, whereas R2 exhibited a substantial increase, reaching nearly 14 Å after 130 ns of simulation. R3 displayed intermediate behavior, reaching approximately 7 Å at 170 ns. Such mobility likely reflects adaptation of the ligand within the binding cavity rather than ligand escape. This behavior is consistent with the structural characteristics of the SARS-CoV-2 3CL^pro^ active site, which consists of a broad and relatively shallow cavity. Furthermore, PG_401 is a large molecule, with a molecular weight of 756.25 Da and a TPSA of 304.21 Å^2^, substantially exceeding the traditional drug-likeness thresholds of 500 Da and 140 Å^2^. Despite these properties and the significant conformational movements observed, the ligand remained bound within the catalytic cavity during all three simulations.

PC1 (Figure S9A) accounted for 16.9% of the total variance, and the simulation evolved from conformations located at negative PC1 values during the early stages to conformations occupying positive PC1 values toward the end of the trajectory, indicating gradual adaptation of the complex to alternative bound states. R2 (Figure S9B) showed the largest conformational transition among the three replicas, with PC1 explaining 52.0% of the variance. The trajectory followed a well-defined U-shaped distribution, connecting temporally distinct conformational clusters and indicating a pronounced collective motion between different yet energetically related conformations. R3 (Figure S9C) displayed an intermediate behavior, with PC1 accounting for 22.9% of the variance. Two major conformational ensembles were observed, linked by intermediate states that illustrate a smooth transition from the initial to the final stages of the simulation. The temporal shift from negative to positive PC1 values suggests a gradual structural reorganization while preserving the integrity of the complex. These results indicate that all three replicas sampled multiple interconnected conformational states through well-organized transitions, reflecting conformational adaptability of the AutoDock Vina-derived complex without compromising the stability of the protein–ligand interaction.

The conformational ensemble sampled across the three replicas was predominantly confined to a broad low-energy basin centered around RMSD ≈ 1.8 Å and Rg ≈ 25.3 Å (Figure S9D). From this global minimum, the landscape extends continuously toward secondary low-energy regions, indicating the presence of multiple energetically accessible substates connected by low free-energy barriers. Such a profile is consistent with a stable binding mode capable of accommodating moderate conformational fluctuations while preserving favorable protein–ligand interactions throughout the simulations.

Binding free energies calculated using the MM/GBSA approach further corroborated the structural analyses. The average binding free energies were −30.74 ± 5.51 kcal/mol for R1, −32.73 ± 5.54 kcal/mol for R2, and −35.73 ± 5.75 kcal/mol for R3. Interestingly, despite the larger conformational variability observed in R2, this trajectory maintained a highly favorable binding energy, indicating that increased flexibility did not compromise ligand affinity. Moreover, R3 exhibited the most favorable binding energy among all simulations, suggesting that the conformational states sampled during this trajectory contributed positively to complex stabilization.

### 2.5. Structural Stability of PL^pro^–Magnoloside I Complexes

#### 2.5.1. Magnoloside I ChemPLP-Docked

The complex PL^pro^–PG_401 was simulated in MD for 200 ns. H-bonds between the ligand and protein BL2loop (residues G266, N267, Y268, Q269, C270, G271) (Figure S10A) and between the ligand and residue Y268 (Figure S10B) were predicted. It was possible to perceive the same profile in the interactions predicted between graphs, with few interactions being predicted in all replicas (R1, R2, and R3), indicating that most interactions were realized with Y268.

R1 had an average of 0.05 H-bonds with the BL2loop, reaching 4 H-bonds at around 15 ns (Figure S10A) and an average of 0.02 interactions with Y268, peaking at 3 H-bonds at around 21 ns (Figure S10B). However, ten unique bonds between the ligand and residues G266, N267, Y268, and Q269 were predicted; of those, nine had occupancy lower than 0.75%, and the last one was a H-bond between the ligand 401 and G266, with occupancy of 2.15%. R2 had an average of 0.02 H-bonds, with a peak of 6 H-bonds in the first 10 ns with the BL2loop (Figure S10A), and 0.01 average interactions with Y268, reaching 5 bonds in the first 10 ns (Figure S10B). There were seven unique bonds between the ligand and residues G266, Y268, and Q269. Six were predicted with occupancy lower than 0.90%, and the last one was a H-bond between the ligand 401 and G266, with occupancy of 0.90%.

The R3 average of H-bonds with the BL2loop was also 0.02, with peak interactions happening before 10 ns, reaching 3 simultaneous H-bonds (Figure S10A), and the average of H-bonds with Y268 was 0.01, peaking at 2 H-bonds (Figures S10B). Ten unique bonds between the ligand and residues G266, Y268, and Q269 were predicted. Nine were predicted with occupancy lower than 0.85%, and the last one was a H-bond between the ligand 401 and G266, with occupancy of 0.85%.

**Figure 7:**
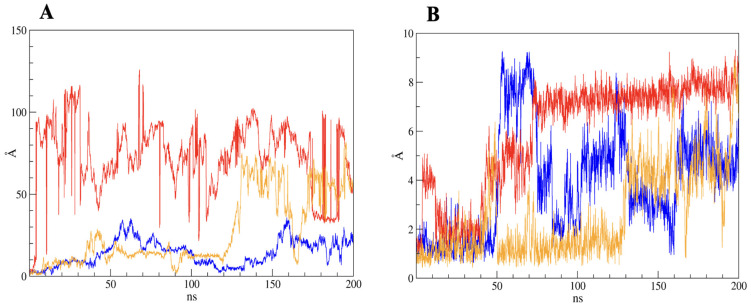
RMSD calculated for PL^pro^ (**A**) BL2 loop and for the (**B**) PG_401 ChemPLP-docked over 200 ns of molecular dynamics simulations. R1 is shown in blue, R2 in red, and R3 in orange.

The RMSD was calculated for the BL2loop and the ligand to observe their movement during the simulation. As previously mentioned, stabilization of the BL2loop creates a physical barrier that stops the protein substrate from interacting with the active site, and therefore, BL2loop stabilization is a very promising strategy [7]. Figure 7A,B show the RMSD calculated for the BL2loop and for the ligand. It is possible to observe a high RMSD for the loop across all replicas, with R1 having an average of 3.884 Å. It is possible to observe a rapid increase in RMSD at around the 50 ns mark, indicating high movement, during the simulation.

R2 had an average RMSD of 5.978 Å, increasing at around 40 ns and again at around 70 ns, remaining high for the rest of the simulation. R3’s RMSD average was 2.577 Å, with an increase in the RMSD values occurring at around 130 ns. This indicates a high movement of the region and therefore a possible extension of the site, allowing the substrate to interact with the active site. The RMSD for the ligand (Figure 7B) is extremely high, with an average of 14.187 Å in R1, 72.889 Å in R2, and 26.573 Å in MD3.

The RMSF calculation reaffirms results obtained for the RMSD, as shown in Figure 7A,B and Table S8. It is possible to observe that BL2loop residues have an increase in the fluctuation (Figure S11A), with residues N267, Y268, and Q269 having values higher than 3 Å in R1, 4 Å in R2, and 2 Å in R3. This translates to a freedom of movement of the middle section of the loop, indicating possible extension of the BL2loop, and therefore, incapacity of the ligand to stabilize the loop.

**Figure 8:**
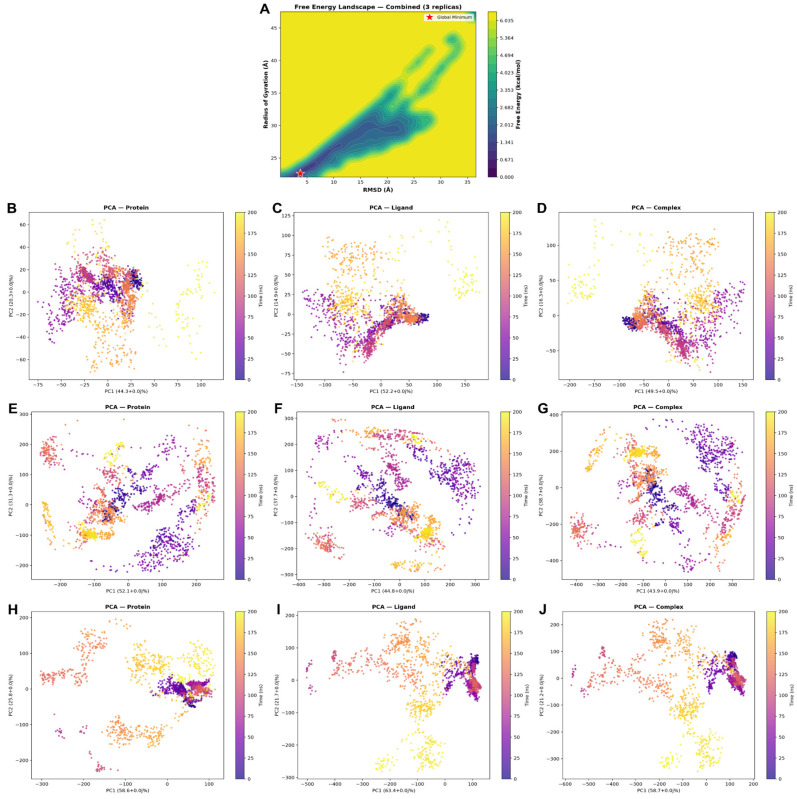
(**A**) Free Energy Landscape (FEL) analysis for the PL^pro^ complex of ChemPLP; (**B**–**J**) PCA of the MD simulation for the protein, ligand, and complex; (**B**–**D**) replicate 1, (**E**–**G**) replicate 2, and (**H**–**J**) replicate 3.

FEL analysis shows that the complex remained mostly compact and structurally similar to the reference structure, with RMSD ≈ 3.5 Å and Rg ≈ 22.6 Å. As the RMSD increases, so does Rg, indicating that any structural drift away from the reference is accompanied by the protein becoming progressively less compact (Figure 8A).

The R1 and R3 PCA for the protein, ligand, and complexes, however, shows that the simulation starts more clustered but ends disperse and far away from the start, indicating possible distinct structural territory. This could reflect partial dissociation, a large binding site rearrangement, or loss of a stable pose. R1 components explain more than 64% of the observed variance for the ligand, protein, and complex, while for R3, more than 79.9% is explained. As for R2, the analysis demonstrates that the system does not appear to be settling into a stable state by the end of 200 ns, as observed by the radial plot, indicating possible partial unfolding, large domain rearrangements, loop excursions, or (in the ligand’s case) significant repositioning/unbinding–rebinding events, especially since the ligand PCA shows similarly extreme, disconnected clustering (Figure 8B–J).

The analysis of the three trajectories’ replicas shows that the ligand was incapable of remaining and stabilizing the BL2loop, allowing its extension and therefore the interaction of the substrate with the active site [50] This can be caused by its size, bigger than the pocket, and, as shown by Gao, 2021 [7], ligands that are too big do not fit in the BL2loop pocket and therefore are incapable of stabilizing the loop, even when they remain interacting, causing an extension of the loop, still allowing the substrate access to the active site.

#### 2.5.2. Magnoloside I GoldScore-Docked 

When compared to ChemPLP, the GoldScore-generated pose exhibited a greater number of H-bonds with the BL2loop and Y268 (Figure S10C,D). However, only R3 showed persistent interactions throughout the simulation, with an average of 1.4 H-bonds formed with the BL2loop and a maximum of 13 simultaneous H-bonds (Figure S10C). Interactions with Y268 were also more frequent in R3, averaging 0.5 H-bonds and reaching up to 4 concurrent interactions at multiple time points during the simulation (Figure S10D). A total of eight unique H-bonds involving residues Y268 and Q269 were predicted. Of these, three exhibited occupancies below 0.30%, whereas three interactions with Q269 showed occupancies of 6.20%, 31.61%, and 45.40%, indicating that Q269 contributed the most persistent contacts with ligand 401. Additionally, two interactions between Y268 and ligand 401 displayed occupancies of 29.50% and 28.70%.

In contrast, R1 and R2 exhibited fewer and less persistent interactions. R1 showed an average of 0.5 H-bonds and 22 unique predicted interactions, of which 20 displayed occupancies below 5.35%, while two interactions with Q269 reached occupancies of 10.20% and 14.50%. Similarly, R2 presented an average of 0.4 H-bonds and 14 interactions, 13 of which had occupancies below 9%, whereas a single interaction with Q269 showed an occupancy of 12.10%. The maximum number of simultaneous H-bonds observed was 8 for R1 and 6 for R2 (Figure S10C). Interactions with Y268 were considerably less frequent in these replicas, with an average of 0.1 H-bonds and peak values of 3 and 4 simultaneous interactions for R1 and R2, respectively (Figure S10D).

**Figure 9:**
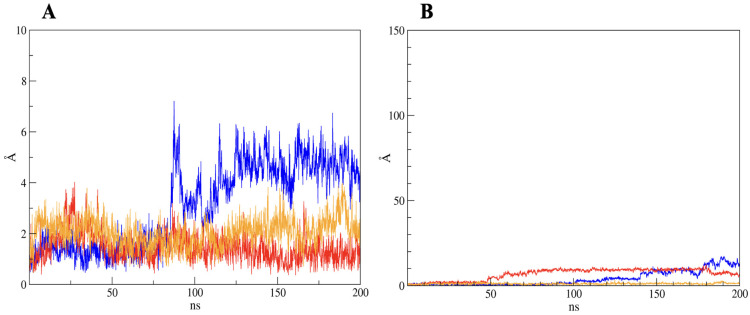
RMSD calculated for PL^pro^ (**A**) BL2 loop and for the (**B**) PG_401 GoldScore-docked over 200 ns of molecular dynamics simulations. R1 is shown in blue, R2 in red, and R3 in orange.

The RMSD for the BL2loop and the ligand (Figure 9A,B) shows that R1 was the most unstable, with the RMSD averaging around 3.1 Å, and peaking at 7 Å around 80 ns (Figure 9A). The ligand RMSD for R1 also shows instability, with an average of 4.1 Å, peaking at 17.17 Å at the 190 ns mark (Figure 9B). The R2 RMSD for the BL2loop was more stable, with an average of 1.46 Å, and the highest point at 4 Å around 25 ns (Figure 9A), as for the ligand, there was a 6.8 Å average and a peak of 12.6 Å around the 180 ns mark (Figure 9B). The R3 ligand was the most stable, with an RMSD average of 1.15 Å, peaking at 3.25 Å (Figure 9B), and for the BL2loop, the average RMSD was 2.0 Å, peaking at 4 Å close to the 190 ns mark (Figure 9A).

The RMSF of the BL2loop shows that of the 3, R1 had the most fluctuation for residues Y268 and Q269 (peaking at 3.2 Å and 3.5 Å respectively), demonstrating some instability of the region. R1 and R2 were very stable, with the calculated RMSF values not crossing the 2 Å threshold for all residues (Figure S11B and Table S8).

**Figure 10:**
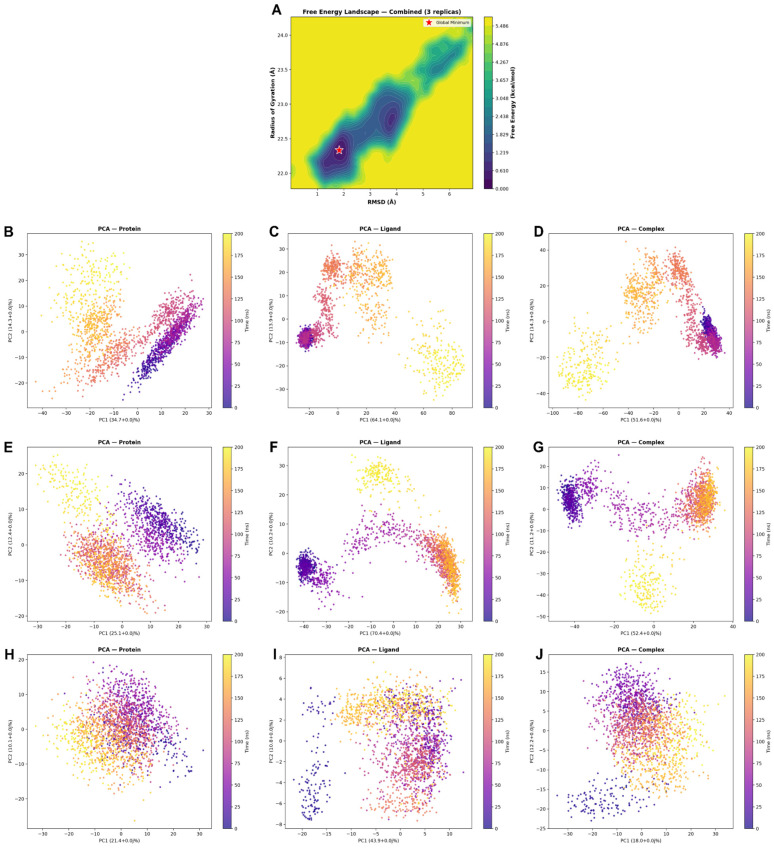
(**A**) Free Energy Landscape (FEL) analysis for the PL^pro^ complex of GoldScore; (**B**–**J**) PCA of the MD simulation for the protein, ligand, and complex; (**B**–**D**) replicate 1, (**E**–**G**) replicate 2, and (**H**–**J**) replicate 3.

FEL analysis shows high levels of compactness and low movements of the structure with RMSD ≈ 2 Å and Rg ≈ 22.4 Å. As with ChemPLP, an RMSD increase results in a concurrent Rg increase, indicating that any structural drift away from the reference is accompanied by the protein becoming progressively less compact (Figure 10A).

The R1 PCA for the protein, ligand, and complexes, however, shows that the simulation starts more clustered and shifts as the simulation progresses. This behavior indicates the presence of conformational transitions throughout the simulation rather than the maintenance of a single dominant conformation. These observations are consistent with the RMSD and RMSF analyses, which suggest increased mobility associated with loop opening. The PC components for the ligand and complex are above 69%; however, for protein, they are 49%. Therefore, a third PC would be needed to explain most of the observed behavior (Figure 10B–D). R2 demonstrates that the system assumed three distinct conformations, with all three structures having a clear pattern of distinct clusters. This suggests that the system sampled three major conformational states throughout the simulation, and considering the pattern is also present on the ligand, as well as the observed RMSD and RMSF values, the ligand stayed in the pocket and stabilized the loop throughout the simulation (Figure 10E–G). The PCs for the ligand and complex are above 63%; however, the first two PCs explain only 37.5% of the protein variance, suggesting a more complex conformational landscape that cannot be fully captured without considering additional principal components. As for R3, all structures remained in a single cluster; although the cluster was relatively broad, the structures remained within a single conformational basin and changed in a coordinated manner, and always did so together (Figure 10H–J). The PCs for the protein, ligand, and complex were 31.5%, 54.7%, and 30.2% respectively.

When analyzing the trajectory for R1, throughout the trajectory the ligand interacts with both the protein and the BL2loop; however, it allows the loop extension [7]. This could be explained by its size; for bigger ligands, even if interacting with the loop, they force it open, potentializing the protein activity [7]. This, however, was not observed during the R2 and 3 trajectories and shows that the ligand stays in the pocket, stabilizing the loop in a way to not allow it to extend.

Thus, these results inform us that this pose of the ligand may be capable of stabilizing the BL2loop in a closed conformation, with all three replicas being predicted with a high number of H-bonds between the ligand and BL2loop, specifically Y268, and R3 realizing the most interactions. This indicates that this pose could be able to correctly inhibit SARS-CoV-2’s PL^pro^.

#### 2.5.3. Magnoloside I AutoDock Vina-Docked

Analysis of the trajectories for the pose generated by AutoDock Vina revealed distinct behaviors across the replicates. In R1, the ligand shifted within the pocket but remained adjacent to the BL2loop, maintaining it in a closed conformation. In R2, the ligand transiently left the pocket but subsequently re-entered, stabilizing the loop upon rebinding. Conversely, in R3, compound PG_401 was unable to remain within the binding pocket, failing to stabilize the BL2loop.

The H-bond prediction for R1 showed that most interactions with the BL2loop were predicted in the first half of the trajectory, with a mean value of 0.3 H-bonds throughout the simulation, with a maximum of 5 simultaneous interactions (Figure S10E); with 12 unique bonds being predicted, 9 had occupancy below 6%. One interaction with N267, Y268, and G266 had 7.70%, 7.25%, and 6.55% respectively. For Y268, the average of H-bonds was 0.13, with a maximum at 3 simultaneous interactions (Figure S10F).

R2’s interactions with the BL2loop were the inverse of R1’s, with most of them in the second half of the simulation, averaging at 0.4, peaking at 9 simultaneous bonds (Figure S10E), with 14 unique bonds being predicted, and 12 had occupancy below 6%. Two interactions with Y268 had 10.85% and 10.00%. Interactions with Y268 followed the same profile, with most interactions in the second half, a mean of 0.23 H-bonds, and a peak at 4 interactions multiple times (Figure S10F).

R3 was predicted with the fewest interactions, which can be explained by the ligand leaving the pocket during the trajectory. The average predicted H-bond interactions with the BL2loop were 0.12, peaking at 6 interactions at around the 75 ns mark (Figure S10E), with 18 unique bonds being predicted, and 16 had occupancy below 2%. Two interactions with N267 had 3.60% and 2.95%, and the average of the interactions with Y268 was 0.02, peaking at 2 simultaneous interactions (Figure S10F).

**Figure 11:**
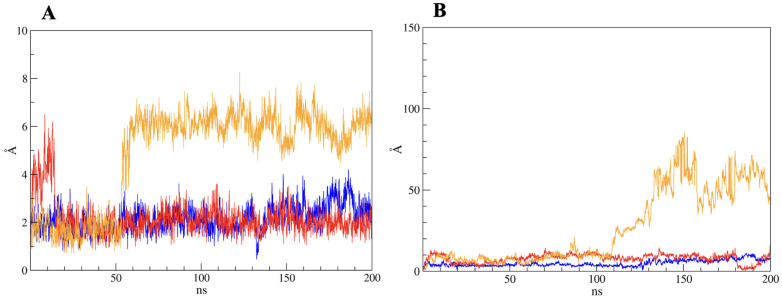
RMSD calculated for PL^pro^ (**A**) BL2 loop and for (**B**) PG_401 AutoDock Vina over 200 ns of molecular dynamics simulations. R1 is shown in blue, R2 in red, and R3 in orange.

The RMSD for the BL2loop and the ligand (Figure 11A,B) shows that R3 was the most unstable, with the RMSD averaging around 4.8 Å, peaking at 8.2 Å around 120 ns (Figure 11A). The ligand RMSD for R3 also shows high instability, with an average of 26.7 Å, peaking at 85 Å at the 150 ns mark (Figure 11B). The R1 and R2 RMSD for the BL2loop were very similar, being stable, with an average of 2.145 Å for R1 and 2.148 Å for R2, and the highest point at 4.19 Å around 190 ns and 6.5 Å at the 15 ns mark respectively (Figure 11A). As for the ligand, the averages differ. The mean RMSD for R1 was 4.9 Å, peaking at 11.2 Å around the 185 ns mark, while for R2, the average RMSD was 7.9, maxing out at 16 Å around 135 ns (Figure 11B).

The RMSF of the BL2loop shows that R3 had the most fluctuation, demonstrating higher instability of the region. For residues N267 and C270, the calculated RMSF values were above 2 Å, while for residues Y268 and Q269, they were above 4.5 Å (Table S8). These values demonstrate the high instability of the region. R2 is more stable, with only the RMSF values for residues Y268 and Q269 being above 2 Å. The R1 RMSF values are all below the 2 Å threshold (Table S8; Figure S11C).

**Figure 12:**
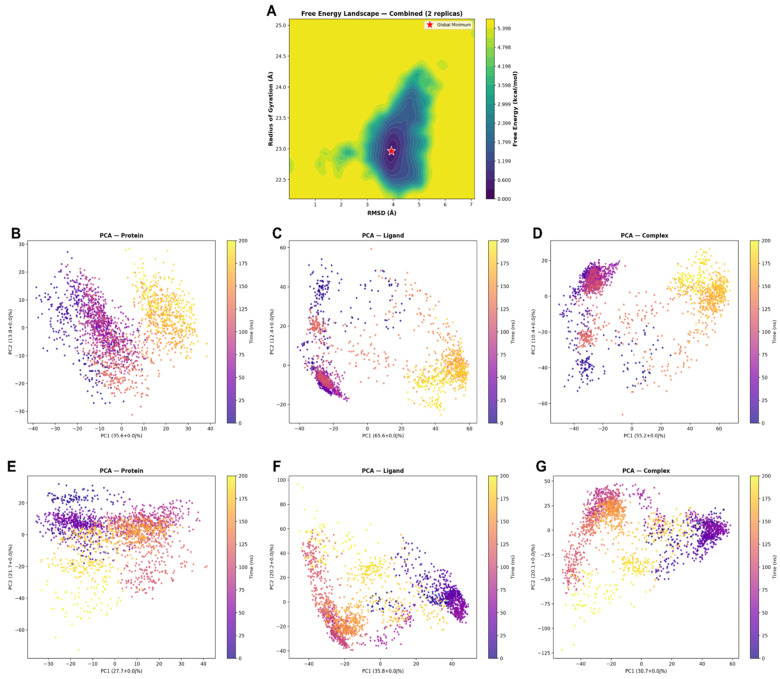
(**A**) Free Energy Landscape (FEL) analysis for the PL^pro^ complex of AutoDock Vina; (**B**–**G**) PCA of the MD simulation for the protein, ligand, and complex; (**B**–**D**) replicate 1 and (**E**–**G**) replicate 2.

FEL analysis showed less compactness relative to the other scoring methods (≈23 Å), with a global minimum at higher structure deviations (≈4 Å), but the maximum deviation reached was at lower RMSD values compared to the other scoring functions (Figure 12A).

The R1 PCA for the protein shows clustered pattern, with two distinct clusters, one for the first half of the simulation and another for the second half. This demonstrates that the protein remained mostly stable during the simulation; however, this stability is only 49.4% explained by PC1 and 2. The ligand and complex, however, have a more spread out clustering, demonstrating instability of the complex, probably due to the ligand either having a lot of movement inside the pocket or even leaving the binding pocket. The calculated PC variances were 78% and 65.6 % respectively. Considering the RMSD and RMSF values, this can be explained by ligand rotation, but not movement away from the pocket, still stabilizing the loop and protein structure (Figure 12B–D).

R2 demonstrates a more clustered graph, with clusters in close proximity for the protein, and for the ligand and complex following 2 clusters. This would indicate that as the simulation progressed, the protein changed from one conformation to another, and this can be observed in the RMSD and RMSF values, as well as H-bonds, with the second half of the simulation having lower RMSD values and more H-bonds between the ligand and the protein (Figure 12E–G). The calculated PC variances were 40.4%, 56%, and 50.8% respectively.

From these results we can infer that BL2loop stabilization might be pose-dependent, with two out of the three replicas being predicted as realizing H-bonds between the ligand and BL2loop, specifically Y268, and R1 and R2 remaining in the pocket throughout the simulation. This indicates that this pose could be able to correctly inhibit SARS-CoV-2’s PL^pro^.

The molecular dynamics simulations highlighted distinct interaction behaviors of PG_401 with SARS-CoV-2’s 3CL^pro^ and PL^pro^ proteases. For 3CL^pro^, PG_401 maintained stable hydrogen bonds with key catalytic residues (E166, Q189, and H41) [48], remaining consistently within the active site. RMSD and RMSF analyses supported its structural stability and moderate mobility, reinforcing its potential as a multitarget inhibitor. In contrast, PL^pro^ simulations revealed greater variability, with ChemPLP and AutoDock Vina poses failing to stabilize the BL2loop, while GoldScore showed more promising results, particularly R2 and R3. Nonetheless, high loop fluctuations and ligand displacement suggest that PG_401’s size may limit its ability to fully inhibit PL^pro^. These findings support PG_401—magnoloside I—as a strong candidate for 3CL^pro^ inhibition, with conditional potential for PL^pro^ depending on the BL2loop dynamics.

### 2.6. In Vitro Inhibition of SARS-CoV-2 r3CL^pro^ and rPL^pro^ by Verbascoside and Calceolarioside B

In vitro studies of the 22 PGs from our initial screening list found no previous reports of their direct inhibition of SARS-CoV-2 proteases. However, several structural derivatives have been reported in the literature to possess such activity. For instance, echinacoside, which lacks the caffeic acid unit present in PG_105 (6‴-*O*-caffeoyl echinacoside), exhibited inhibitory activity against 3CL^pro^ with an IC_50_ of 6.97 ± 0.20 μM [12].

Regarding PG_376 (Forsythoside J), previous studies have evaluated other compounds of the same family (Forsythosides A, B, E, H, and I) against 3CL^pro^, showing IC_50_ values ranging from 4.49 to 9.09 μM. Among these, forsythoside H is particularly notable (IC_50_ = 4.69 ± 0.31 μM), differing from PG_376 only by the substitution of the outer α-rhamnopyranosyl unit with a β-xylopyranosyl unit [12].

Furthermore, magnoloside B differs from PG_401 (magnoloside I) by the presence of a caffeoyl unit and a rhamnose linked to glucose, whereas PG_401 contains a coumaroyl unit and an apiose linked to glucose. Previous works have shown that magnoloside B inhibits 3CL^pro^ with an IC_50_ of 7.78 ± 0.16 μM [12].

These documented structural correlations strongly support the predictive reliability of our in silico model, highlighting the intrinsic potential of the phenylethanoid glycoside scaffold to bind and inhibit SARS-CoV-2 proteases. To experimentally confirm this biological potential, we conducted in vitro enzymatic assays using commercially available PGs: calceolarioside B (PG_013) and verbascoside, a representative anchor compound in this class.

**Figure 13:**
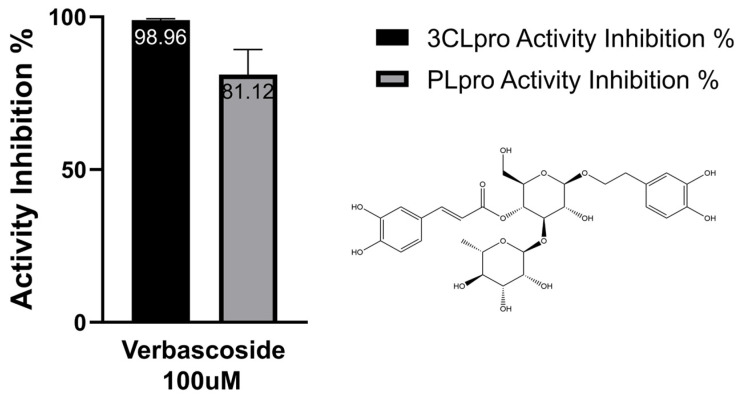
Inhibitory effect of verbascoside (100 μM) on SARS-CoV-2 3CL^pro^ and PL^pro^ proteolytic activity, determined by the FRET assay. Data are presented as mean ± SD (*n* = 3).

The inhibition kinetics of verbascoside against 3CL^pro^ and PL^pro^ reinforced the high potential of this metabolite class, demonstrating strong inhibition against both proteases (Figure 13). In the study by [12], results consistent with our findings were reported for 3CL^pro^ inhibition (100 μM: 98.89%; 10 μM: 95.98%; IC_50_: 4.83 ± 0.03 μM).

**Figure 14:**
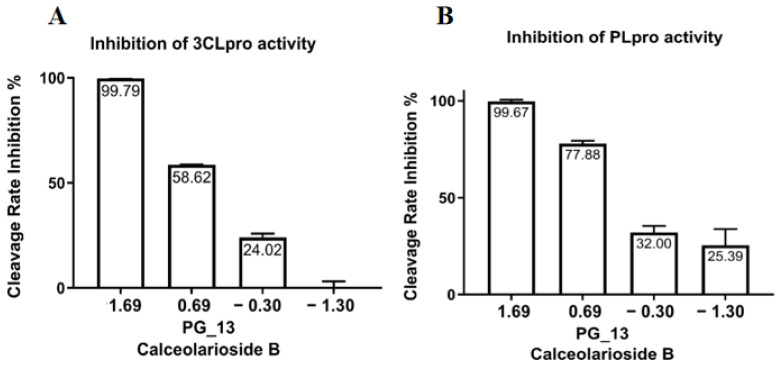
Dose–response curves of PG_13 (calceolarioside B) against SARS-CoV-2 3CL^pro^ (**A**) and PL^pro^ (**B**), assessed by FRET assay. PG_13 was tested over a concentration range of 50–0.05 uM. Data are presented as mean of inhibition ± SD (*n* = 3).

Dose–response curves of PG_013 (calceolarioside B) against 3CL^pro^ and PL^pro^ are shown in Figure 14. Calceolarioside B inhibited more than 90% of the activity of both proteases at the highest concentration tested (50 μM). After a 10-fold reduction in concentration, PG_13 still inhibited more than 50% of the enzymatic activity, with a particularly pronounced effect against PL^pro^, for which inhibition remained above 70%. At lower concentrations, however, the inhibitory activity against both enzymes decreased to below 30%.

Despite the structural similarities within this class of compounds, minor functional-group modifications can significantly alter their pharmacological and safety profiles, posing toxicity risks, as observed with PG_013. However, this localized risk does not diminish the overall therapeutic impact of the phenylethanoid glycoside scaffold. Instead, it underscores the critical importance of our methodology: leveraging rigorous in silico toxicity predictions to filter out candidates with mutagenic or hepatotoxic potential, thereby prioritizing only the safest and most effective derivatives (such as PG_144, PG_146, PG_383, PG_401, and PG_542) for future COVID-19 drug development.

## 3. Methods and Materials

### 3.1. Creation of a Phenylethanoid Glycoside (PG) Dataset

A database of phenylethanoids described in the literature was created based on four review manuscripts. Jimenez et al. reviewed the period from the first isolation of a phenylethanoid in 1950 until 1992 [51]. Fu et al. described compounds identified from 1997 to 2007 [27], while Xue et al. described those from 2009 to 2015 [52]. Finally, Wu et al. presented phenylethanoids identified from 1993 to 1997, from 2007 to 2009, and from 2016 to 2020 [53]. A total of 567 structures were drawn using ChemDraw (version Pro.12) software or MarvinSketch server (https://marvinjs-demo.chemaxon.com/latest/demo.html) (accessed on 4 July 2023) and revised for literature inconsistencies, such as the same compound being published with two different trivial names or different compounds being published with the same trivial name.

### 3.2. Molecular Docking Protocol

To determine the protonation state, we used PROPKA 3.0 software via the PDB2PQR [54] server (http://server.poissonboltzmann.org/pdb2pqr) (accessed on 4 July 2023) with the AMBER force field to calculate the pKa values of amino acid residues of 3CL^pro^ and PL^pro^ at pH 7.4. We compared the predicted values to those reported in the literature on the amino acid titration curve. The ligands NNA and GRL0617, which are inhibitors of 3CL^pro^ and PL^pro^, respectively, were prepared using OpenBabel [55] (version 3.1.0) functions.

The redocking of 3CL^pro^ and PL^pro^ proteases was performed using AutoDock Vina [56] (version 1.1.2) and the GOLD Suite [57] (version 2022.3.0). The exhaustiveness was set to 100 for AutoDock Vina, and the ChemPLP and GoldScore score functions were used for the GOLD Suite. The binding site parameters were set to a radius of 10 Å from the center of the ligands for both proteases. The redocking results were evaluated using the Root Mean Square Deviation (RMSD), calculated with OpenBabel. The RMSD was calculated by comparing the first docking pose with the conformation of the ligands found in the crystallized protein structure. Maestro [58] (version 13.1.141) was used for further analysis of the redocking results. The amino acid residues of concern in 3L^pro^ are H41, C145, and E166 and in PL^pro^ are N267, Y268, and Q269.

Molecular docking simulations were performed using three scoring functions defined in the redocking parameters to evaluate in silico interactions between the proteases and the 567 glycosylated phenylethanoids. The simulations used AutoDock Vina and GOLD Suite, which includes the ChemPLP and GoldScore scoring functions. The virtual screening results were then reclassified using the penalty score. The penalty score is a consensus score that reclassifies compounds based on the sum of their ranking positions in each simulation: AutoDock Vina, GOLD/ChemPLP, and GOLD/GoldScore. These values are then divided by the total of 548 molecules. The penalty score ranges from zero to one, and a better compound classification in the final consensus score is indicated by values closer to zero. Finally, the compounds that showed the most promising interaction with 3CL^pro^ and PL^pro^ SARS-CoV-2 were selected. The ligand–receptor interaction of this final complex was analyzed in the same way as in the redocking step.

### 3.3. In Silico ADMETox Prediction

The pharmacokinetic properties of molecules were analyzed using ADMETlab 2.0 [59]. The analyzed properties included Molecular Weight (MW), Number of Hydrogen Bond Acceptors (nHA), Number of Hydrogen Bond Donors (nHD), Number of Rotatable Bonds (nRot), Topological Polar Surface Area (TPSA), Logarithm of the n-octanol/water distribution coefficient (LogP), Pan Assay Interference compounds (PAINS), and prediction of intestinal cellular absorption (Caco-2 permeability). The work predicts the substrate capability (Pgp-sub) or inhibition (Pgp-inh) of P-glycoprotein, Human Intestinal Absorption (HIA), penetration of the Blood–Brain Barrier (BBB), and Fraction Unbound in plasma (Fu). Toxicity parameters such as human hepatotoxicity (H-HT), drug-induced liver injury (DILI), and Ames test results for mutagenicity, carcinogenicity, and respiratory toxicity were used as filters to select compounds.

### 3.4. Molecular Dynamics Protocol

Molecular dynamics (MD) was performed to assess the stability of the compound’s pose in the catalytic pocket over a period of 200 nanoseconds (ns). The simulations were performed using the AMBER 2022 and AmberTools2022 software package [60]. For both 3CL^pro^ (PDBid: 6XQT) and PL^pro^ (PDBid: 7JRN), the protein file was protonated to pH 8.0 to mimic the pH conditions of the in vitro assays, using the H^++^ web server (http://newbiophysics.cs.vt.edu/H++/) (accessed on 20 September 2023) [61,62]. Ligand preparation was performed using the Antechamber tool. PL^pro^ complex preparation was performed using the MCPB.py tool [63] and GAMESS-US [64] to properly simulate the zinc atom present in the protein and its effect on protein motility.

The force fields used were ff19SB [65], gaff2 [66], and Optimal Point Charge (OPC) [67] for water. Solvation was performed in a truncated octahedron box with 12.0 Å from the box edge. The salt molarity was calculated using the method described by Machado (2020) [68] to neutralize the system and was set at 0.15 M. The relaxation was made with 1000 cycles of steepest descent. The temperature was set at 310 K. Final simulations were performed for 200 ns with Monte Carlo Barostat. SHAKE was used to suppress hydrogen bonding. The time step was set to 0.001. This production step was performed in triplicate. Hydrogen bonds and the RMSD were calculated using VMD. The Root-Mean-Square Fluctuation (RMSF) was calculated using the CPPTRAJ module present in AMBER.

At last, the MD simulations were carried out in triplicate (R1, R2, and R3) for each scoring function used in molecular docking (ChemPLP, GoldScore, and AutoDock Vina).

The principal component analysis (PCA) and free energy landscape (FEL) analyses were performed using the PepIntProt (version 4.0) [69]. For PCA, molecular dynamics trajectories were aligned to a reference structure, and the covariance matrix of atomic positional fluctuations was diagonalized to identify the principal components describing the dominant collective motions of the system. Conformational sampling was visualized by projecting the trajectories onto the first two principal components (PC1 and PC2). FELs were generated using the RMSD and radius of gyration (Rg) as reaction coordinates. Free energy values were calculated, considering 310K, from the probability distribution of sampled conformations according to the Boltzmann relation, G = −*k_B_T*ln*P*, allowing the identification of global and local energy minima, characterization of energetically favorable conformational states, and evaluation of the stability and conformational plasticity of the protein–ligand complexes. The software also enables the extraction of representative structures from the lowest-energy basins and the combined analysis of multiple simulation replicas.

Binding free energies were estimated using the MM/GBSA approach implemented in the MMPBSA.py module of Amber22. Calculations were performed using snapshots extracted from the equilibrated portion of each 200 ns molecular dynamics trajectory, considering frames 1500 to 2000 at intervals of two frames. The Generalized Born implicit solvent model GB-Neck2 was employed (igb = 8) with a salt concentration of 0.150 M.

### 3.5. FRET-Based In Vitro Inhibition Assays of SARS-CoV-2 r3CL^pro^ and rPL^pro^

Recombinant SARS-CoV-2 3CL^pro^ and PL^pro^ proteases, expressed in *E. coli* BL21(DE3)pLysS and BL21(DE3) cells, respectively, were employed in a Fluorescence Resonance Energy Transfer (FRET) assay to assess the proteolytic activity inhibition. Verbascoside (phyproof^®^ Reference Substance, Merck^®^, ≥90.0% purity by HPLC) was tested at 100 μM. For the dose–response curve, calceolarioside B (phyproof^®^ Reference Substance, Merck^®^) with a purity of ≥95.0% (HPLC) was tested in triplicate at 50, 5, 0.5, and 0.05 μM.

For 3CL^pro^, the enzyme concentration was maintained at 1.5 μM, and the mixture was incubated with 5 mM NaCl, 20 mM Tris-HCl (pH 8.0), and 5 mM DTT (Merck, Darmstadt, Germany—97%, CAS 3483-12-3) for 15 min at 37 °C. For PL^pro^, a similar protocol was followed, except that the enzyme concentration was set at 1 μM and the reaction buffer contained 150 mM NaCl, 20 mM Tris-HCl (pH 8.0), and 5 mM DTT. The reaction was initiated by adding the substrate (50 μM final concentration, dissolved in DMSO).

The customized fluorogenic substrates used were DABCYL-Ala-Val-Leu-Gln↓Ser-Gly-Phe-Arg-Lys-EDANS for 3CL^pro^ and DABCYL-Ala-Leu-Lys-Gly↓Gly-Lys-Iso-Val-EDANS for PL^pro^ (GenScript, Piscataway, NJ, USA, >95% purity). Fluorescence was measured using a SpectraMax M5 multi-mode microplate reader (Molecular Devices, San Jose, CA, USA). The EDANS fluorescence emission was monitored at λexc = 330 nm and λem = 490 nm at 37 °C for 45 min.

Fluorescence data (RFU) were converted to substrate cleavage-specific activity based on a previously calculated fluorescence conversion factor (FEC) for the EDANS-DABCYL fluorophore pair. The maximum enzyme activity was measured using the vehicle (DMSO), and these values were used to calculate the inhibition percentages of the test compounds. Additionally, the fluorescence profiles of the test compounds were assessed under the same assay conditions (dissolved in DMSO, buffer, and substrate). This step was essential to confirm the reliability of the inhibition results by ruling out autofluorescence. Enzyme activity was also evaluated using the commercial inhibitors GC-376 for 3CL^pro^ and GRL-0617 for PL^pro^ (Figure S3).

## 4. Conclusions

In summary, our study aimed to identify PGs from species used in traditional medicine, predominantly Chinese, as potential inhibitors of SARS-CoV-2’s 3CL^pro^ and PL^pro^ enzymes. Through in silico analysis, we identified 22 PGs with promising inhibitory activity against both enzymes, based on their multitarget capabilities and low penalty scores.

Additionally, toxicity assessments identified five PGs—4-*cis*-*p*-coumaroyl mussatioside, 4-feruloyl mussatioside, fucatoside C, magnoloside I, and lagotiside C (PG_144, PG_146, PG_383, PG_401, and PG_542, respectively)—as non-toxic and non-carcinogenic, thus making them the most promising candidates for further investigation.

Receptor–ligand interaction studies provided insights into the binding affinity and specificity of selected PGs with 3CL^pro^ and PL^pro^. Molecular dynamics simulations highlighted the stability and interaction dynamics of the PG–enzyme complexes. Notably, PG_401 demonstrated consistent and stable interactions with key residues of 3CL^pro^ across all MD replicas, supporting its potential as a 3CL^pro^ inhibitor. Against PL^pro^, however, interactions were pose-dependent, with only the GoldScore pose achieving BL2loop stabilization in two of three replicas. Thus, while PG_401 exhibits multitarget binding capability in silico, its activity against PL^pro^ appears conditional and may require structural optimization to achieve consistent inhibition.

Despite this behavior, the PCA and FEL analyses consistently demonstrated that the PG_401 complexes remained confined to energetically favorable conformational states for both 3CL^pro^ and PL^pro^, indicating that the ligand forms structurally stable complexes with both targets throughout the simulations. These findings reinforce the multitarget potential of PG_401, although additional optimization may be required to achieve more consistent inhibition of PL^pro^.

Furthermore, the experimental evaluation of PG_13 (calceolarioside B) revealed strong inhibitory activity against both viral proteases. PG_13 inhibited 3CL^pro^ by 99.79% and PL^pro^ by 99.67% at 50 μM, while maintaining measurable inhibitory activity over the entire concentration range tested. Although in silico toxicity screening predicted a potential mutagenic risk for PG_13, this result should be interpreted with caution and investigated further.

Taken together, these results demonstrate that phenylethanoid glycosides represent a valuable source of antiviral scaffolds against SARS-CoV-2. In particular, PG_401 exhibited favorable pharmacological and structural properties, stable binding profiles, and robust energetic behavior against both viral proteases, whereas PG_13 showed remarkable experimental inhibition of 3CL^pro^ and PL^pro^. These findings support the continued investigation of both compounds as possible hits for the development of novel anti-coronavirus therapeutics.

## Data Availability

The original contributions presented in this study are included in the article/Supplementary Materials. Further inquiries can be directed to the corresponding authors.

## Supplementary Materials

The following supporting information can be downloaded at: https://www.mdpi.com/article/10.3390/ph19071126/s1, Table S1. PGs predicted as top candidates for SARS-CoV-2 protease inhibition; 22 PGs with penalty scores ≤0.3 against 3CL^pro^ and PL^pro^ identified through in silico screening. Figure S1. Ligand poses obtained from the redocking experiment, compared with the crystallographic ligand NNA (PDB ID: 6XQT), magenta colored. (A) Pose generated using GOLD–ChemPLP. (B) Pose generated using GOLD–GoldScore. (C) Pose generated using AutoDock Vina. Figure S2. Ligand poses obtained from the redocking experiment, compared with the crystallographic ligand TTT (PDB ID: 7JRN), magenta pink. (A) Pose generated using GOLD–ChemPLP. (B) Pose generated using GOLD–GoldScore. (C) Pose generated using AutoDock Vina. Table S2. Docking score obtained using the GOLD–ChemPLP scoring function and 3CL^pro^ residues interacting with the PG. Table S3. Docking score obtained using the GOLD–GoldScore scoring function and 3CL^pro^ residues interacting with the PG. Table S4. Docking score obtained using AutoDock Vina and 3CL^pro^ residues interacting with the PG. Table S5. Docking score obtained using the GOLD–ChemPLP scoring function and PL^pro^ residues interacting with the PG. Table S6. Docking score obtained using the GOLD–GoldScore scoring function and PL^pro^ residues interacting with the PG. Table S7. Docking score obtained using AutoDock Vina and PL^pro^ residues interacting with the PG. Figure S3. H-bond interactions between the 3CL^pro^-PG 401 complex over 200 ns of molecular dynamics simulations. A, B, and C correspond to R1, R2, and R3, respectively, based on the pose obtained using the ChemPLP docking scoring function. D, E, and F represent R1, R2, and R3 pose obtained with the GoldScore function. G, H, and I illustrate R1, R2, and R3 derived from the AutoDock Vina. Each plot depicts the time-dependent number of hydrogen bonds between PG_401 and SARS-CoV-2 3CL^pro^. Figure S4. GOLD ChemPLP-derived 3CL^pro^-PG401 complex during 200 ns of molecular dynamics simulations. In all panels, R1 is shown in blue, R2 in red, and R3 in orange. (A) RMSD of the 3CL^pro^-PG401 complex. (B) RMSD of the 3CL^pro^ binding-pocket residues. (C) RMSF profile of key binding-pocket residues, with residues involved in ligand interactions highlighted. Figure S5. PCA of the 3CL^pro^-PG401 complex obtained from the ChemPLP-generated binding pose. PCA projections along the PC1 and PC2 for the three independent replicas: (A) R1, (B) R2, and (C) R3. The color scale represents simulation time (0–200 ns), illustrating the conformational sampling of the complex throughout the trajectories. Figure S6. GOLD GoldScore-derived 3CL^pro^-PG401 complex during 200 ns of molecular dynamics simulations. In all panels, R1 is shown in blue, R2 in red, and R3 in orange. (A) RMSD of the 3CL^pro^-PG401 complex. (B) RMSD of the 3CL^pro^ binding-pocket residues. (C) RMSF profile of key binding-pocket residues, with residues involved in ligand interactions highlighted. Figure S7. PCA of the 3CL^pro^-PG401 complex obtained from the GoldScore-generated binding pose. PCA projections along the PC1 and PC2 for the three independent replicas: (A) R1, (B) R2, and (C) R3. The color scale represents simulation time (0–200 ns), illustrating the conformational sampling of the complex throughout the trajectories. (D) FEL of the 3CL^pro^-PG401 complex projected onto RMSD and Rg, with the global minimum indicated by the red star. Figure S8. GOLD AutoDock Vina-derived 3CL^pro^-PG401 complex during 200 ns of molecular dynamics simulations. In all panels, R1 is shown in blue, R2 in red, and R3 in orange. (A) RMSD of the 3CL^pro^-PG401 complex. (B) RMSD of the 3CL^pro^ binding-pocket residues. (C) RMSF profile of key binding-pocket residues, with residues involved in ligand interactions highlighted. Figure S9. PCA of the 3CL^pro^-PG401 complex obtained from the AutoDock Vina-generated binding pose. PCA projections along the PC1 and PC2 for the three independent replicas: (A) R1, (B) R2, and (C) R3. The color scale represents simulation time (0–200 ns), illustrating the conformational sampling of the complex throughout the trajectories. (D) FEL of the 3CL^pro^-PG401 complex projected onto RMSD and Rg, with the global minimum indicated by the red star. Figure S10. H-bond interactions between the PL^pro^-PG 401 complex over 200 ns of molecular dynamics simulations. In all graphs R1 is colored in blue, R2 in red, and R3 in orange. A and B correspond to H-bonds predicted between BL2loop and only residue Y268, respectively, based on the pose obtained using the ChemPLP docking scoring function. C and D correspond to H-bonds predicted between BL2loop and only residue Y268, respectively, obtained with the GoldScore function. E and F correspond to H-bonds predicted between BL2loop and only residue Y268, respectively, derived from the AutoDock Vina. Each plot depicts the time-dependent number of hydrogen bonds between PG_401 and SARS-CoV-2 PL^pro^. Table S8. RMSF values in angstromns for PL^pro^’s BL2loop residues. All three replicates of each scoring method. Figure S11. RMSF of the residues forming the BL2loop of PL^pro^ over 200 ns of molecular dynamics simulations. In all graphs, R1 is shown in blue, R2 in red, and R3 in orange. (A) GOLD–ChemPLP PL^pro^–PG_401 complex. (B) GOLD–GoldScore PL^pro^–PG_401 complex. (C) AutoDock Vina PL^pro^–PG_401 complex.

## Abbreviations

Coronavirus disease 2019	COVID-19
Hydrogen bonds	H-bonds
Interferon Stimulated Gene 15	ISG15
3-chymotrypsin-like protease	3CL^pro^
Molecular Weight	MW
Papain-Like protease	PL^pro^
Severe Acute Respiratory Syndrome Coronavirus 2	SARS-CoV-2
Virtual Screening	VS
Molecular Dynamics	DM
