# Supplementary material for: Computational Evaluation of Multitarget Capabilities of Phenylethanoid Glycosides Against SARS-CoV-2’s 3CLpro and PLpro
Source: Pharmaceuticals (Basel). 2026 Jul 21;19(7):1126. doi: 10.3390/ph19071126 (PMC13414898; doi:10.3390/ph19071126)
Supplement: Supplementary file 1 [file pharmaceuticals-19-01126-s001.zip › pharmaceuticals-4346725-supplementary.pdf]

**Table S1.** PGs predicted as top candidates for SARS-CoV-2 protease inhibition. 22 PGs with penalty scores ≤0.3 against 3CL$^{pro}$ and PL$^{pro}$ identified through *in silico* screening.

<table>
<tr><td>Code</td><td>Compound</td><td>Reference</td><td>Molecular Weight (Da)</td></tr>
<tr><td>PG_005</td><td>Lippiarubelloside A</td><td>(compound 42) – [16]</td><td>786.733</td></tr>
<tr><td colspan="4">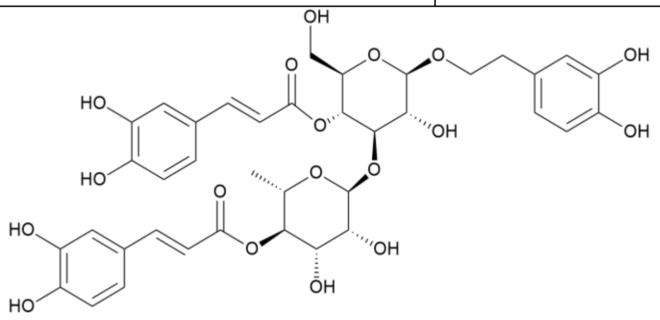</td></tr>
<tr><td>Code</td><td>Compound</td><td>Reference</td><td>Molecular Weight (Da)</td></tr>
<tr><td>PG_013</td><td>Calceolariside B</td><td>(compound 5) – [46]</td><td>480.464</td></tr>
<tr><td colspan="4">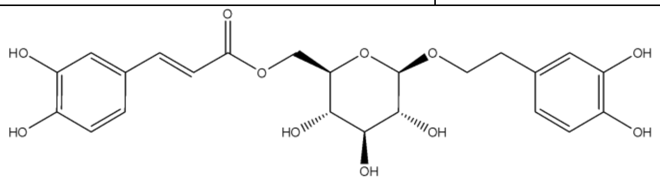</td></tr>
<tr><td>Code</td><td>Compound</td><td>Reference</td><td>Molecular Weight (Da)</td></tr>
<tr><td>PG_028</td><td>2',3',4',6'- Treta-O-galloyl salidroside</td><td>(compound 20) – [46]</td><td>908.725</td></tr>
<tr><td colspan="4">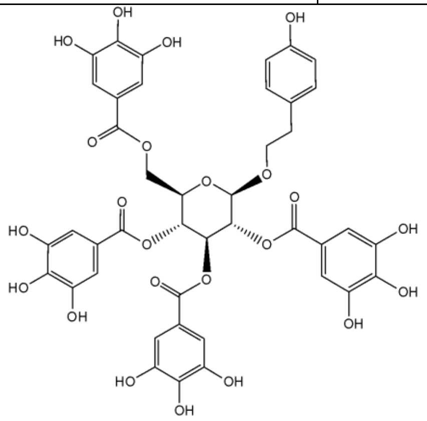</td></tr>
<tr><td>Code</td><td>Compound</td><td>Reference</td><td>Molecular Weight (Da)</td></tr>
<tr><td>PG_105</td><td>6'''-O-caffeoyl echinacoside</td><td>(compound 100) – [46]</td><td>948.874</td></tr>
</table>

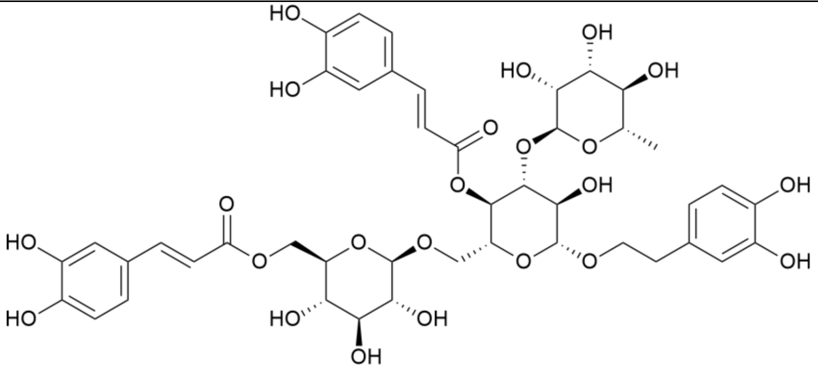

| Code | Compound | Reference | Molecular Weight (Da) |
|---|---|---|---|
| PG_118 | Myricoside Api (1'''◎ 3'') Rha | (compound 113) – [46] | 756.704 |

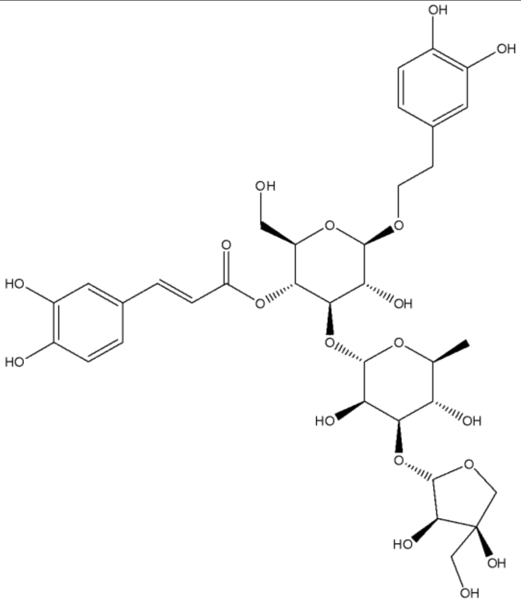

| Code | Compound | Reference | Molecular Weight (Da) |
|---|---|---|---|
| PG_130 | Phlinoside C Rha(1'''--> 2'') Rha | (compound 122) – [46] | 770.731 |

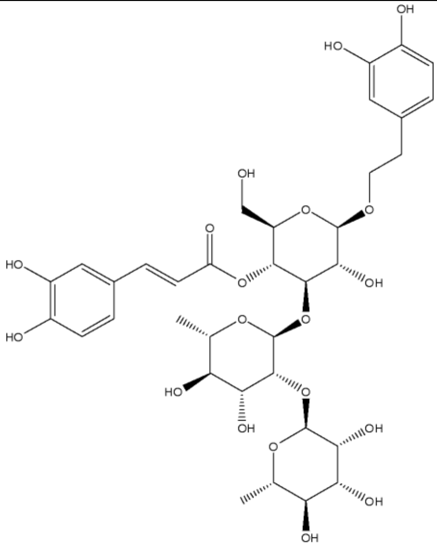

| Code | Compound | Reference | Molecular Weight (Da) |
|---|---|---|---|
| PG_144 | 4-cis-p-coumaroyl mussatioside | (compound 140) – [46] | 724.706 |

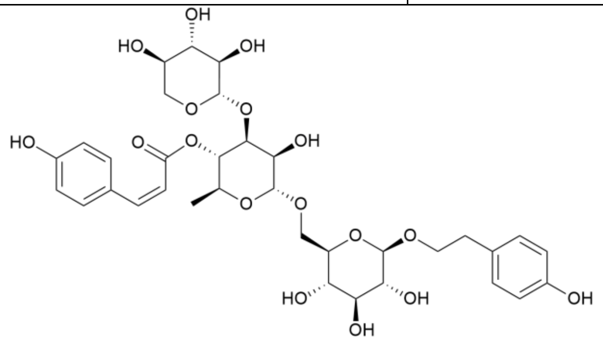

| Code | Compound | Reference | Molecular Weight (Da) |
|---|---|---|---|
| PG_146 | 4-feruloyl mussatioside | (compound 142) – [46] | 754.732 |

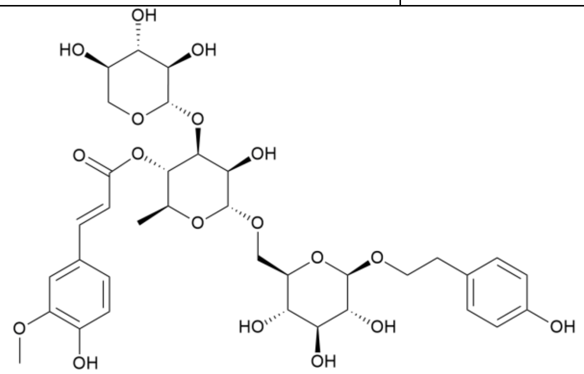

| Code | Compound | Reference | Molecular Weight (Da) |
|---|---|---|---|
| PG_169 | Lamiusides B | (compound 11) – [47] | 802.773 |

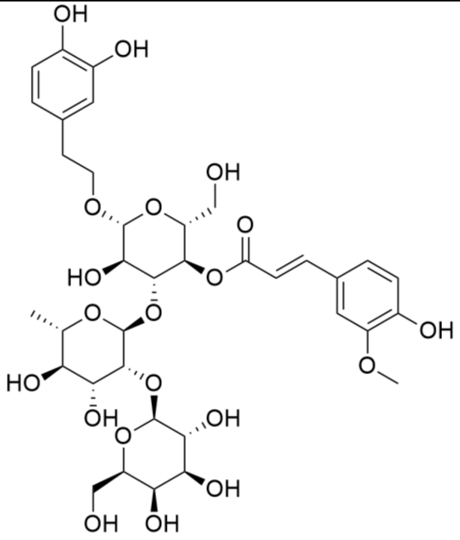

| Code | Compound | Reference | Molecular Weight (Da) |
|---|---|---|---|
| PG_182 | Myricoside - 3''''-O-methylether | (compound 24) – [47] | 770.731 |

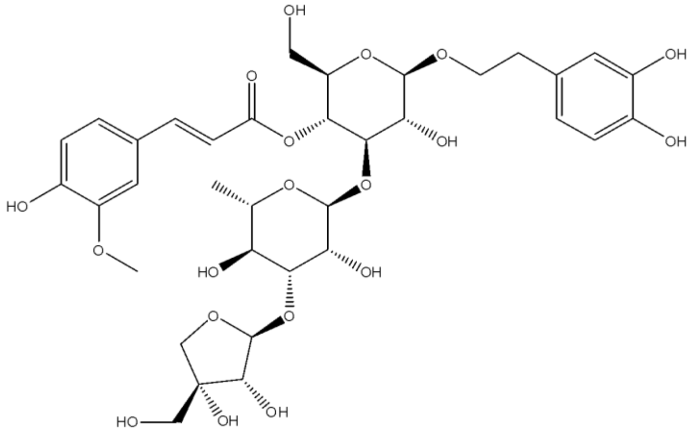

| Code | Compound | Reference | Molecular Weight (Da) |
|---|---|---|---|
| PG_196 | Ligurobustosides N | (compound 39) – [47] | 770.731 |

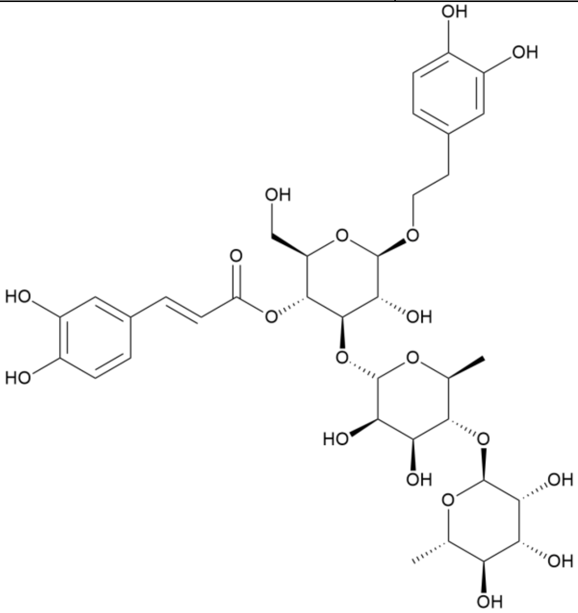

| Code | Compound | Reference | Molecular Weight (Da) |
|---|---|---|---|
| PG_325 | Rossicaside F | (compound 171) – [47] | 830.783 |

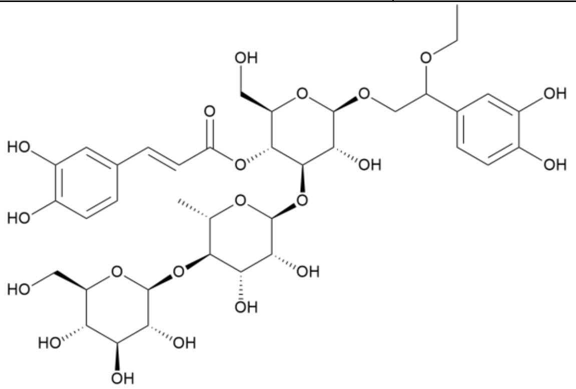

| Code | Compound | Reference | Molecular Weight (Da) |
|---|---|---|---|

| | | | |
|---|---|---|---|
| PG_336 | Safghanoside E | (compound 182) – [47] | 796.769 |

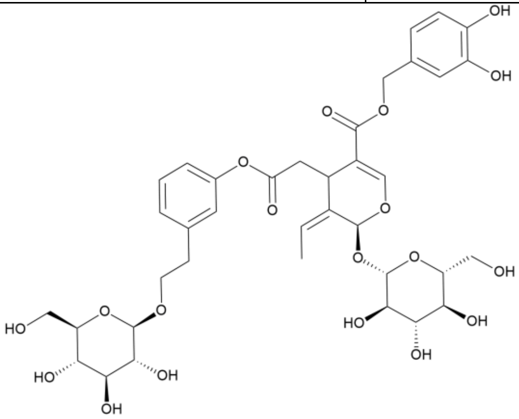

| Code | Compound | Reference | Molecular Weight (Da) |
|---|---|---|---|
| PG_367 | Purpureaside D | (compound 23) – [28] | 638.617 |

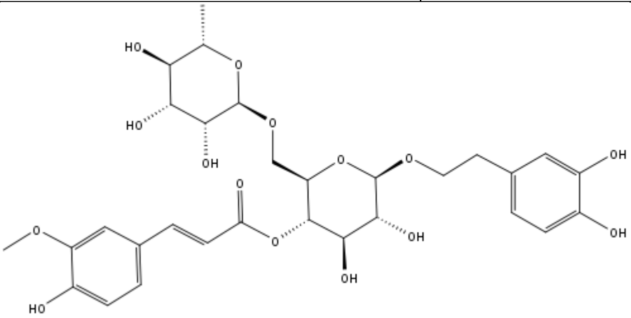

| Code | Compound | Reference | Molecular Weight (Da) |
|---|---|---|---|
| PG_371 | 2-(3,4-Dihydroxyphenyl)-2-oxoethyl-O-α -L-rhamnopyranosyl- (1⊚6)-(4-O-caffeoyl)- β -D-glucopyranoside | (compound 27) – [28] | 624.589 |

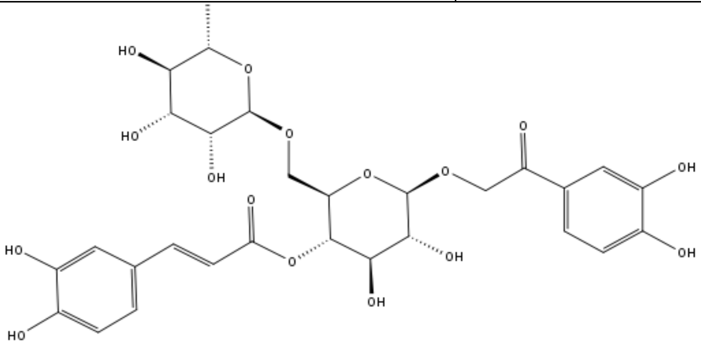

| Code | Compound | Reference | Molecular Weight (Da) |
|---|---|---|---|
| PG_376 | Forsythoside J | (compound 32) – [28] | 610.563 |

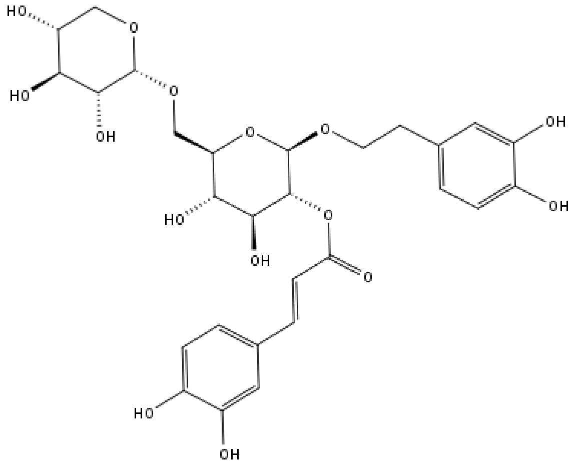

| Code | Compound | Reference | Molecular Weight (Da) |
|---|---|---|---|
| PG_383 | Fucatoside C | (compound 39) – [28] | 744.693 |

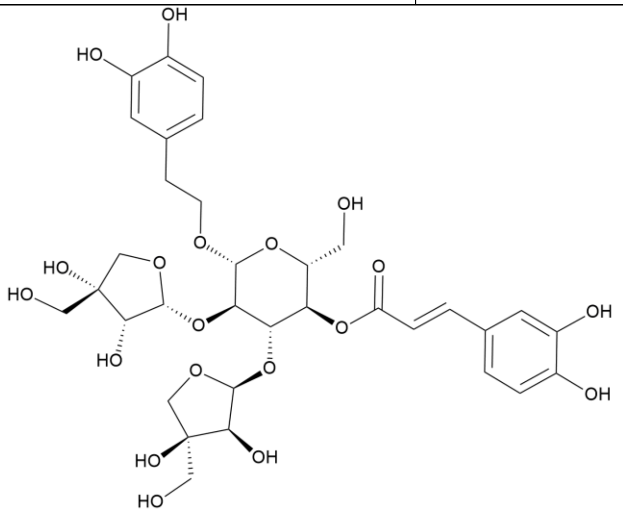

| Code | Compound | Reference | Molecular Weight (Da) |
|---|---|---|---|
| PG_388 | β-(3,4-Dihydroxyphenyl) ethyl-6-O-E-caffeoyl-O-[β -D-apiofuranosyl-( 1®2)]-β- D-glucopyranoside | (compound 44) – [28] | 610.563 |

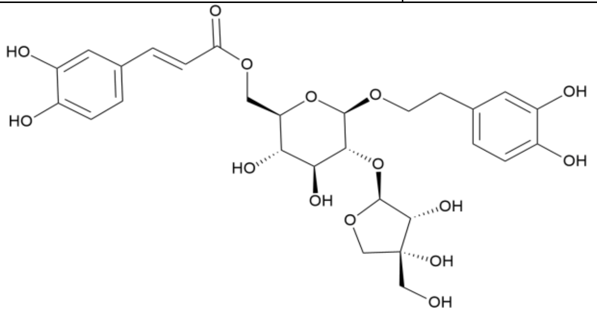

| Code | Compound | Reference | Molecular Weight (Da) |
|---|---|---|---|
| PG_401 | Magnoloside I | (compound 57) – [28] | 756.704 |

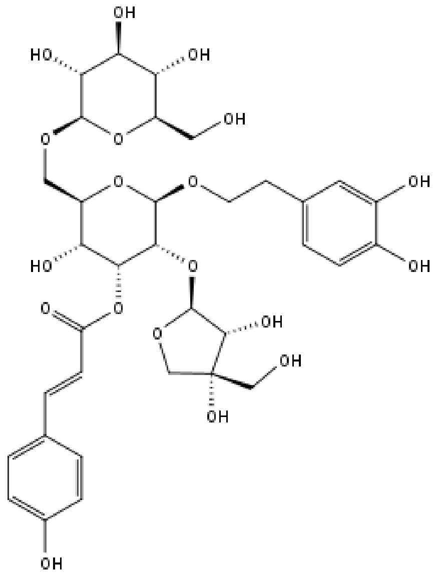

| Code | Compound | Reference | Molecular Weight (Da) |
|---|---|---|---|
| PG_489 | Digiviridifloroside | (compound 27) –[16] | 816.759 |

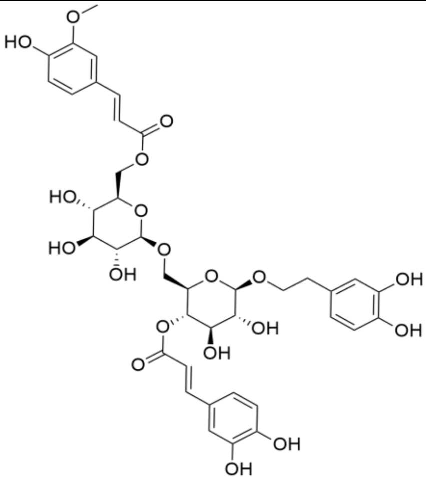

| Code | Compound | Reference | Molecular Weight (Da) |
|---|---|---|---|
| PG_525 | Macrophylloside F | (compound 65) –[16] | 640.589 |

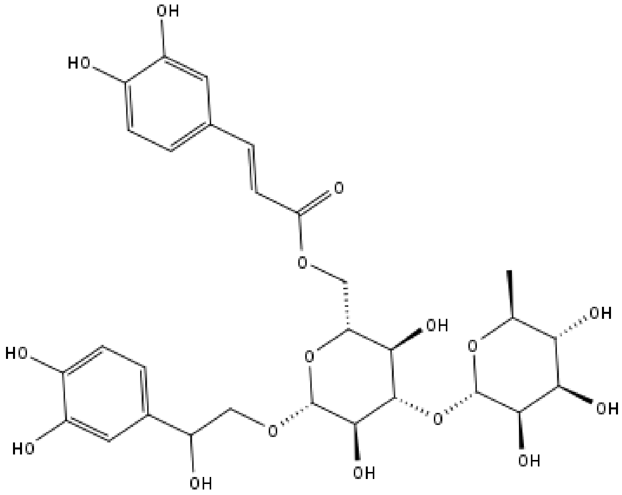

| | | | |
|---|---|---|---|
| Code | Compound | Reference | Molecular Weight (Da) |
| PG_542 | Lagotiside C | (compound 84) –[16] | 786.73 |
| 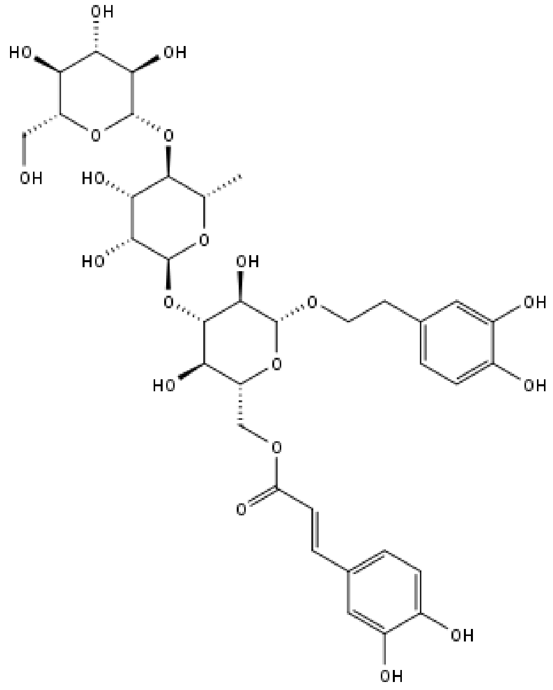 | | | |

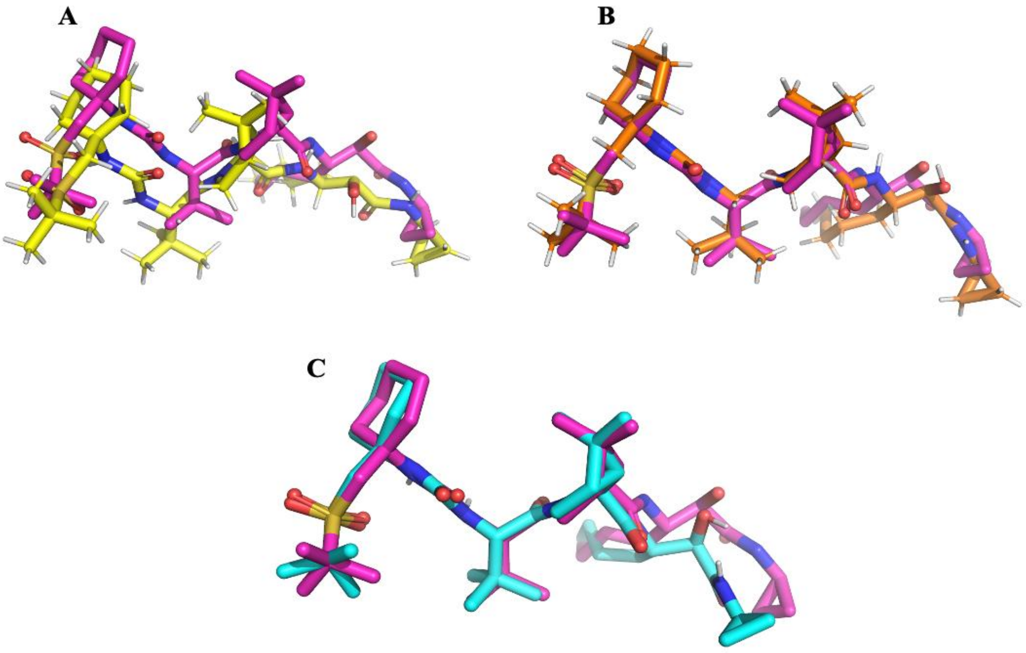


**Figure S1.** Ligand poses obtained from the redocking experiment, compared with the crystallographic ligand NNA (PDB ID: 6XQT), magenta colored. (**A**) Pose generated using GOLD–ChemPLP. (**B**) Pose generated using GOLD–GoldScore. (**C**) Pose generated using AutoDock Vina.

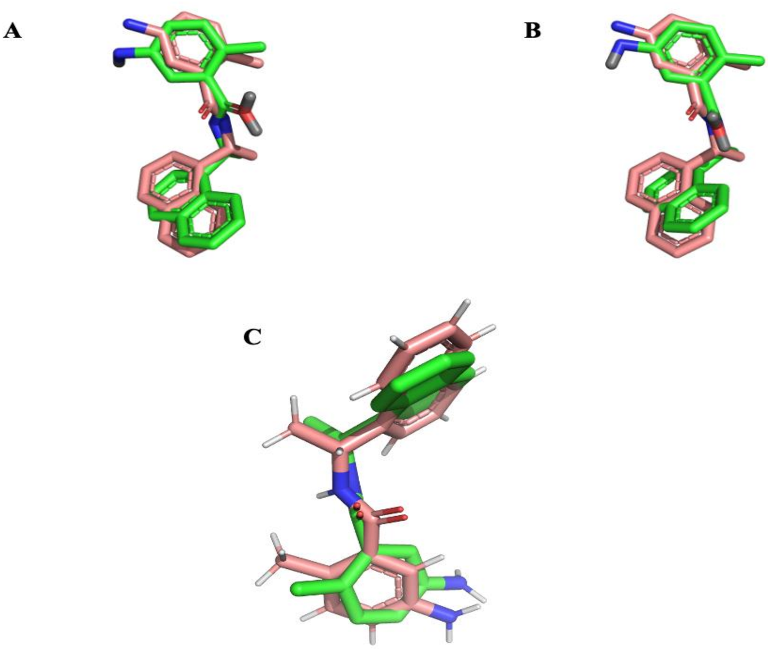


**Figure S2.** Ligand poses obtained from the redocking experiment, compared with the crystallographic ligand TTT (PDB ID: 7JRN), magenta pink. (**A**) Pose generated using GOLD–ChemPLP. (**B**) Pose generated using GOLD–GoldScore. (**C**) Pose generated using AutoDock Vina

**Table S2.** Docking score obtained using the GOLD–ChemPLP scoring function and $3CL^{pro}$ residues interacting with the PG.

| ChemPLP | | | | |
|---|---|---|---|---|
| Compound ID | Docking score | Type of Interactions | Residue interacting | Distance (Å) |
| 388 | 98.73 | Hydrogen bonds | E166 | 2.08 |
| | | | L141 | 2.1 |
| | | | L141 | 1.92 |
| | | | S144 | 2.77 |
| | | | T24 | 2.02 |
| | | | T26 | 2.03 |

| | | | | |
|---|---|---|---|---|
| | | | Q192 | 2.11 |
| 105 | 111.79 | | Q192 | 2.12 |
| | | | E166 | 2.08 |
| | | | H164 | 1.82 |
| | | | L141 | 1.66 |
| | | | C145 | 2.25 |
| | | | T26 | 2.0 |
| | | | T24 | 2.04 |
| 401 | 97.86 | | T26 | 2.08 |
| | | | E166 | 1.96 |
| | | | E166 | 2.31 |
| | | | E166 | 2.11 |
| | | | L167 | 2.07 |
| | | | Q192 | 2.11 |
| | | | Q189 | 1.69 |
| | | | L141 | 2.1 |
| | | | C145 | 2.6 |
| 5 | 99.77 | | S46 | 2.32 |
| | | | E166 | 2.09 |

| | | | | |
|---|---|---|---|---|
| | | | E166 | 1.89 |
| | | | H164 | 1.99 |
| | | | Q192 | 2.1 |
| | | | T24 | 1.81 |
| 383 | 107.29 | | T24 | 1.98 |
| | | | T26 | 2.1 |
| | | | T26 | 2.02 |
| | | | D187 | 2.0 |
| | | | F140 | 2.11 |
| | | | Q192 | 1.99 |
| 525 | 93.3 | | Q192 | 1.95 |
| | | | Q192 | 2.1 |
| | | | E166 | 1.65 |
| | | | E166 | 1.88 |
| | | | L141 | 2.37 |
| | | | T24 | 2.08 |
| 118 | 108.49 | | T24 | 2.16 |
| | | | T26 | 2.11 |
| | | | C145 | 2.25 |

| | | | | |
|---|---|---|---|---|
| | | | S144 | 2.48 |
| | | | L141 | 1.97 |
| | | | Q189 | 1.96 |
| | | | E166 | 2.07 |
| | | | T190 | 2.2 |
| | | | Q192 | 2.33 |
| 130 | 101.35 | | Q192 | 2.09 |
| | | | Q189 | 1.61 |
| | | | H41 | 2.1 |
| | | | G143 | 1.72 |
| | | | N142 | 2.11 |
| | | | E166 | 2.01 |
| | | | H164 | 2.11 |
| 489 | 94.28 | | Q192 | 2.1 |
| | | | E166 | 1.79 |
| | | | Q189 | 1.66 |
| | | | L141 | 2.02 |
| 325 | 98.78 | | T192 | 1.92 |
| | | | R188 | 2.09 |

| | | | | |
|---|---|---|---|---|
| | | | E166 | 2.27 |
| | | | L141 | 2.09 |
| | | | N142 | 1.9 |
| | | | T26 | 1.65 |
| 371 | 100.84 | | Q192 | 2.01 |
| | | | T26 | 2.08 |
| | | | T26 | 1.75 |
| | | | E166 | 1.95 |
| | | | E166 | 2.13 |
| 144 | 98.22 | | Q102 | 2.5 |
| | | | T190 | 1.85 |
| | | | Q189 | 2.63 |
| | | | T24 | 2.06 |
| | | | G143 | 2.26 |
| | | | N142 | 2.37 |
| | | | L141 | 2.03 |
| 336 | 100.88 | | Q192 | 2.11 |
| | | | Q192 | 1.79 |
| | | | A191 | 1.78 |

| | | | | |
|---|---|---|---|---|
| | | | T190 | 2.11 |
| | | | H164 | 2.05 |
| | | | H164 | 2.08 |
| 28 | 90.09 | | Q192 | 2.1 |
| | | | E166 | 1.86 |
| | | | L141 | 2.09 |
| | | | L141 | 1.41 |
| | | | N142 | 2.17 |
| 542 | 91.49 | | G143 | 2.8 |
| | | | N142 | 2.27 |
| | | | N142 | 2.39 |
| | | | T190 | 1.7 |
| | | | E166 | 1.89 |
| | | | N119 | 2.13 |
| | | | N119 | 2.07 |
| 196 | 101.17 | | T26 | 1.96 |
| | | | H164 | 2.1 |
| | | | E166 | 2.08 |
| | | | E166 | 1.92 |

| | | | | |
|---|---|---|---|---|
| | | | Q192 | 1.9 |
| | | | Q192 | 2.09 |
| | | | Q189 | 1.69 |
| 376 | 97.88 | | E166 | 2.05 |
| | | | E1,66 | 2.06 |
| | | | H164 | 2.24 |
| | | | L141 | 2.02 |
| | | | L141 | 1.97 |
| | | | Q192 | 1.83 |
| | | | Q192 | 2.02 |
| 367 | 92.25 | | E166 | 2.11 |
| | | | E166 | 1.73 |
| | | | T26 | 2.12 |
| | | | T26 | 2.07 |
| | | | Q189 | 1.83 |
| | | | Q189 | 1.92 |
| | | | Q192 | 2.0 |
| 146 | 93.32 | | Q192 | 2.05 |
| | | | Q192 | 2.1 |

| | | | | |
|---|---|---|---|---|
| | | | E166 | 2.07 |
| | | | Q189 | 2.04 |
| 169 | 91.21 | | F140 | 1.93 |
| | | | N142 | 2.11 |
| | | | T26 | 1.92 |
| | | | T190 | 1.82 |
| | | | Q189 | 2.11 |
| | | | R188 | 2.0 |
| | | | E166 | 1.88 |
| 182 | 105.26 | | L141 | 1.62 |
| | | | N142 | 2.17 |
| | | | T24 | 1.96 |
| | | | T24 | 1.94 |
| | | | E166 | 2.34 |
| | | | R188 | 2.02 |
| | | | R188 | 2.01 |
| | | | Q189 | 2.1 |
| 13 | 94.79 | | H164 | 2.09 |
| | | | Q192 | 1.94 |

| | | | | |
|---|---|---|---|---|
| | | | Q192 | 2.02 |
| | | | F140 | 1.71 |
| | | | N142 | 2.18 |

**Table S3.** Docking score obtained using the GOLD–GoldScore scoring function and 3CL$^{pro}$ residues interacting with the PG.

| GoldScore | | | | |
|---|---|---|---|---|
| Compound ID | Docking score | Type of Interactions | Residue interacting | Distance (Å) |
| 388 | 77.39 | Hydrogen bonds | G143 | 2.09 |
| | | | SER1B | 2.64 |
| | | | R188 | 2.06 |
| 105 | 55.66 | | T26 | 1.83 |
| | | | Q189 | 2.25 |
| | | | Q192 | 2.05 |
| | | | L141 | 1.78 |
| 401 | 97.86 | | E166 | 2.46 |
| | | | V186 | 2.16 |
| | | | M49 | 2.77 |
| 5 | 61.09 | | L167 | 2.43 |
| 383 | 95.85 | | C145 | 1.73 |

| | | | | |
|---|---|---|---|---|
| | | | E166 | 1.92 |
| 525 | 55.73 | | E166 | 2.39 |
| | | | R188 | 2.66 |
| 118 | 48.11 | | T26 | 2.23 |
| | | | C145 | 1.98 |
| | | | L141 | 2.43 |
| | | | E166 | 1.89 |
| | | | T190 | 2.14 |
| 130 | 89.31 | | T190 | 2.49 |
| | | | E166 | 1.86 |
| | | | E166 | 2.62 |
| | | | L167 | 2.74 |
| | | | T26 | 2.08 |
| 489 | 76.4 | | C145 | 2.36 |
| | | | L141 | 1.45 |
| 325 | 87.52 | | E166 | 2.0 |
| | | | C145 | 2.22 |
| | | | Q189 | 2.77 |
| 371 | 76.68 | | T190 | 1.69 |

| | | | | |
|---|---|---|---|---|
| | | | Q189 | 2.3 |
| | | | T26 | 1.52 |
| 144 | 87.6 | | E166 | 1.71 |
| | | | E166 | 2.31 |
| | | | Q189 | 2.8 |
| 336 | 82.7 | | H41 | 2.16 |
| | | | T26 | 1.47 |
| | | | E166 | 2.35 |
| | | | R188 | 2.31 |
| | | | Q192 | 2.72 |
| 28 | 77.75 | | E166 | 1.8 |
| 542 | 89.26 | | E166 | 2.39 |
| 196 | 81.38 | | E166 | 1.44 |
| | | | F140 | 2.51 |
| 376 | 85.25 | | Q192 | 2.3 |
| | | | E166 | 2.2 |
| 367 | 87.04 | | C145 | 2.17 |
| | | | G143 | 1.99 |
| | | | T26 | 2.01 |

| | | | | |
|---|---|---|---|---|
| 146 | 69.71 | | N142 | 1.91 |
| | | | T26 | 2.61 |
| | | | Q189 | 2.76 |
| 169 | 78.67 | | F140 | 2.05 |
| | | | Q189 | 2.58 |
| | | | M49 | 2.31 |
| | | | V186 | 2.7 |
| 182 | 79.81 | | L141 | 2.28 |
| | | | T26 | 1.83 |
| | | | R188 | 1.68 |
| 13 | 76.47 | | D187 | 2.52 |
| | | | Q192 | 2.4 |

**Table S4.** Docking score obtained using AutoDock Vina and 3CL$^{pro}$ residues interacting with the PG.

| AutoDock Vina | | | | |
|---|---|---|---|---|
| Compound ID | Docking score | Type of Interactions | Residue interacting | Distance (Å) |
| 388 | -9.1 | Hydrogen bonds | E166 | 2.34 |
| 105 | -8.9 | | E166 | 2.09 |
| | | | E166 | 2.53 |
| | | | P168 | 2.67 |

| | | | | |
|---|---|---|---|---|
| | | | R188 | 2.23 |
| | | | F140 | 1.89 |
| 401 | -8.9 | | E166 | 2.57 |
| | | | E166 | 2.17 |
| | | | L141 | 2.14 |
| 5 | -8.9 | | G143 | 2.35 |
| 383 | -9.8 | | T26 | 2.39 |
| | | | R188 | 1.94 |
| | | | Q192 | 2.06 |
| 525 | -9.1 | | N142 | 2.27 |
| | | | T190 | 2.11 |
| 118 | -8.8 | | L141 | 2.13 |
| | | | N142 | 2.1 |
| | | | C145 | 2.43 |
| | | | E | 2.23 |
| | | | Q192 | 2.06 |
| | | | T190 | 2.6 |
| | | | T26 | 2.7 |
| | | | T190 | 2.14 |

| | | | | |
|---|---|---|---|---|
| 130 | -8.9 | | N142 | 2.56 |
| | | | T26 | 2.36 |
| 489 | -9 | | E166 | 2.41 |
| 325 | -8.8 | | L141 | 2.69 |
| | | | E166 | 2.59 |
| | | | E166 | 1.86 |
| | | | Q192 | 2.07 |
| 371 | -9.1 | | E166 | 1.69 |
| | | | R188 | 2.3 |
| 144 | -9.4 | | G143 | 1.98 |
| | | | F140 | 2.38 |
| 336 | -9.2 | | T190 | 2.28 |
| | | | Q192 | 2.23 |
| | | | N142 | 2.04 |
| | | | N142 | 2.72 |
| | | | G143 | 2.36 |
| | | | C145 | 2.55 |
| | | | M49 | 2.19 |
| | | | E166 | 2.35 |

| | | | | |
|---|---|---|---|---|
| 28 | -8.8 | | F3B | 1.84 |
| | | | G138 | 2.24 |
| | | | D289 | 2.35 |
| | | | D187 | 2.08 |
| 542 | -9 | | N142 | 2.27 |
| | | | T190 | 2.11 |
| 196 | -8.9 | | Q192 | 2.14 |
| | | | E166 | 2.07 |
| | | | E166 | 2.47 |
| | | | E166 | 2.18 |
| | | | G143 | 2.19 |
| 376 | -8.9 | | T26 | 2.39 |
| | | | R188 | 1.94 |
| | | | L141 | 2.06 |
| 367 | -9.4 | | Q192 | 2.08 |
| | | | G143 | 2.43 |
| | | | N142 | 2.15 |
| | | | E166 | 2.07 |
| | | | E166 | 2.54 |

| | | | | |
|---|---|---|---|---|
| 146 | -8.8 | | E166 | 2.11 |
| | | | E166 | 1.98 |
| | | | L141 | 1.83 |
| 169 | -9.3 | | Q192 | 2.39 |
| 182 | -8.9 | | E166 | 2.39 |
| | | | Q192 | 2.47 |
| | | | F140 | 2.41 |
| 13 | -8.9 | | E166 | 1.97 |
| | | | Q192 | 1.83 |

**Table S5.** Docking score obtained using the GOLD–ChemPLP scoring function and PL$^{pro}$ residues interacting with the PG.

| ChemPLP | | | | |
|---|---|---|---|---|
| Compound ID | Docking score | Type of Interactions | Residue interacting | Distance (Å) |
| 144 | | Hydrogen bonds | L162<br>D164 | 1.67<br>1.73 |
| 146 | | | K157<br>L162<br>D164<br>E167<br>Q269 | 2.12<br>1.64 & 1.87<br>1.95 & 2.12<br>1.88<br>1.69 & 2.21 |

| | | | | |
|---|---|---|---|---|
| 383 | | | L162<br>D164<br>R166<br>Q269<br>Y273<br>T301 | 1.68<br>2.14<br>2.20<br>1.97<br>2.35<br>1.95 |
| 401 | | | L162<br>Y264<br>N267<br>Y268<br>Q269 | 2.06 & 2.07<br>2.28<br>2.23<br>2.02<br>1.63 & 2.08 & 2.19 |
| | | | | |
| 542 | | | K157<br>D164<br>G266 | 2.23<br>2.16<br>2.17 |

**Table S6.** Docking score obtained using the GOLD–GoldScore scoring function and PL$^{pro}$ residues interacting with the PG.

| GoldScore | | | | |
|---|---|---|---|---|
| Compound ID | Docking score | Type of Interactions | Residue interacting | Distance |
| 144 | | Hydrogen bonds | G163<br>G266<br>Q269 | 2.61<br>2.27<br>2.12 |
| 146 | | Hydrogen bonds | E167<br>Q269<br>D302 | 1.61<br>2.18<br>1.53 & 2.35 |
| | | Short distance non-bonded contacts | D164 | 1,12 |

| | | | | |
|---|---|---|---|---|
| 383 | | Hydrogen bonds | D164<br>Y273 | 1.54 & 2.35<br>2.00 |
| | | | | |
| | | Short distance non-bonded contacts | Q269<br>T301 | 1.51<br>1.42 |
| 401 | | Hydrogen bonds | K157<br>E161<br>D164<br>E167<br>Y268<br>Q269<br>T301 | 1.98<br>2.23<br>1.51<br>1.67 & 2.68<br>1.72<br>1.62<br>2.55 |
| | | | | |
| 542 | | Hydrogen bonds | E167<br>T301 | 1.61<br>2.09 |
| | | Short distance non-bonded contacts | D164<br>Y264 | 1.43<br>1.48 |

**Table S7.** Docking score obtained using AutoDock Vina and PL$^{pro}$ residues interacting with the PG.

| AutoDock Vina | | | | |
|---|---|---|---|---|
| Compound ID | Docking score | Type of Interactions | Residue interacting | Distance |

| | | | | |
|---|---|---|---|---|
| 144 | | Hydrogen bonds | Q269 | 2,4 |
| 146 | | | NA | NA |
| 383 | | | R166<br>Y264<br>Q269<br>Y273 | 2.31<br>2.47<br>1.96<br>2.02 |
| 401 | | | R166<br>P248<br>Y268<br>Y273 | 2.00<br>2.63<br>2.36<br>2.20 |
| | | | | |
| 542 | | | L162 | 2,42 |

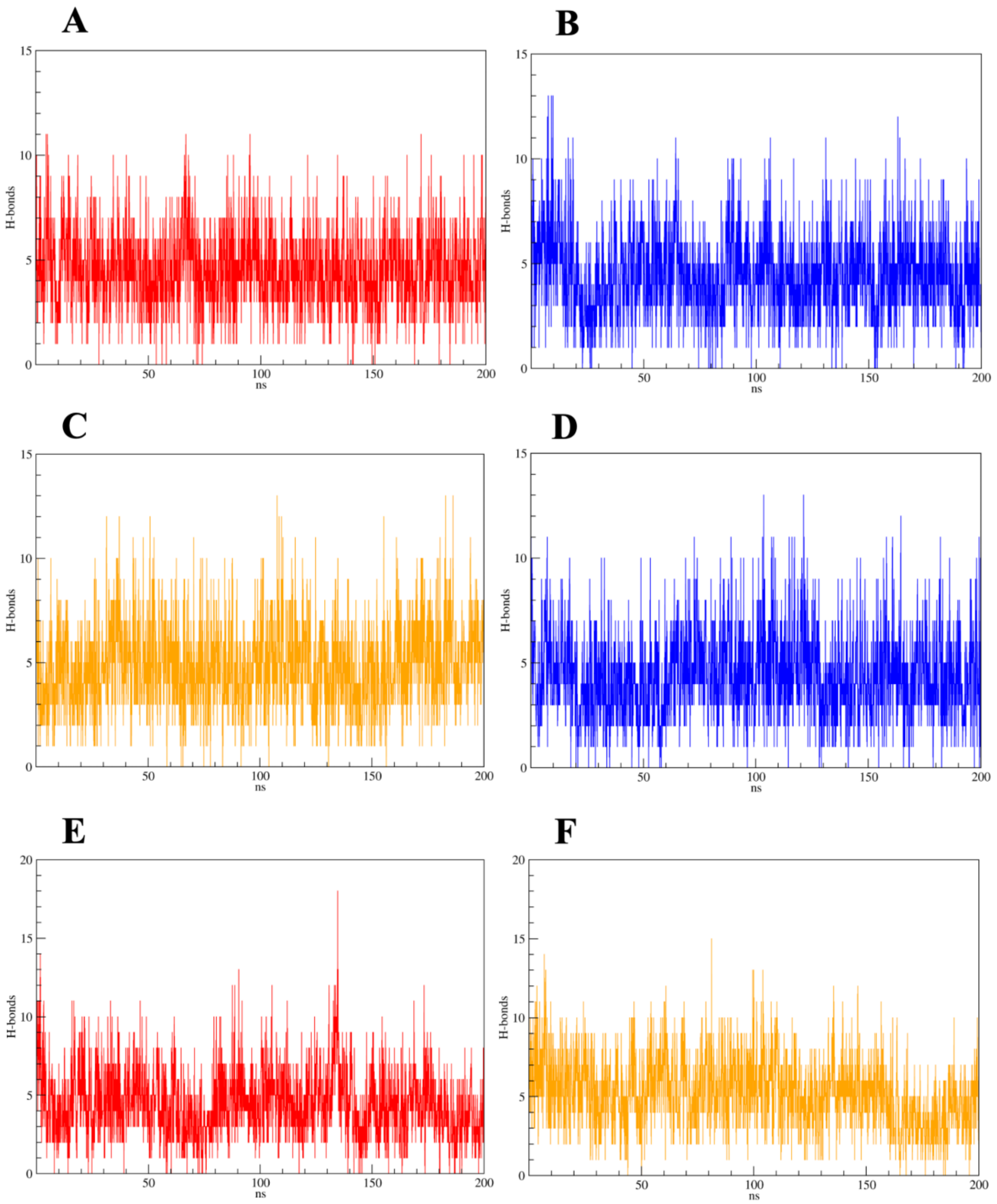

A
B
C
D
E
F
H-bonds
ns
0
5
10
15
20
50
100
150
200

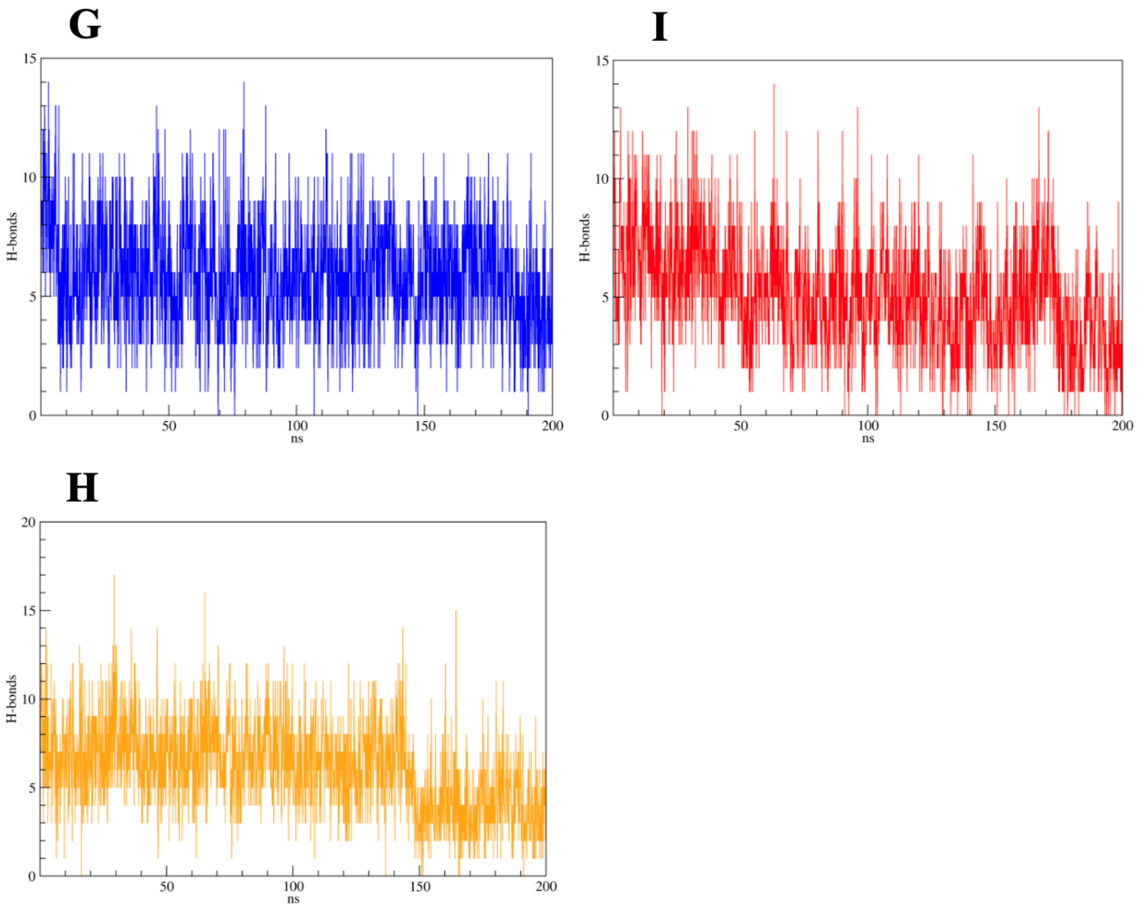


**Figure S3.** H-bond interactions between the $3CL^{pro}$-PG 401 complex over 200 ns of molecular dynamics simulations. S1. **A**, **B**, and **C** correspond to R 1, 2, and 3, respectively, based on the pose obtained using the ChemPLP docking scoring function. S1. **D**, **E**, and **F** represent R 1, 2, and 3 pose obtained with the GoldScore function. S1. G, H, and I illustrate R 1, 2, and 3 derived from the AutoDock Vina. Each plot depicts the time-dependent number of hydrogen bonds between PG_401 and SARS-CoV-2 $3CL^{pro}$.

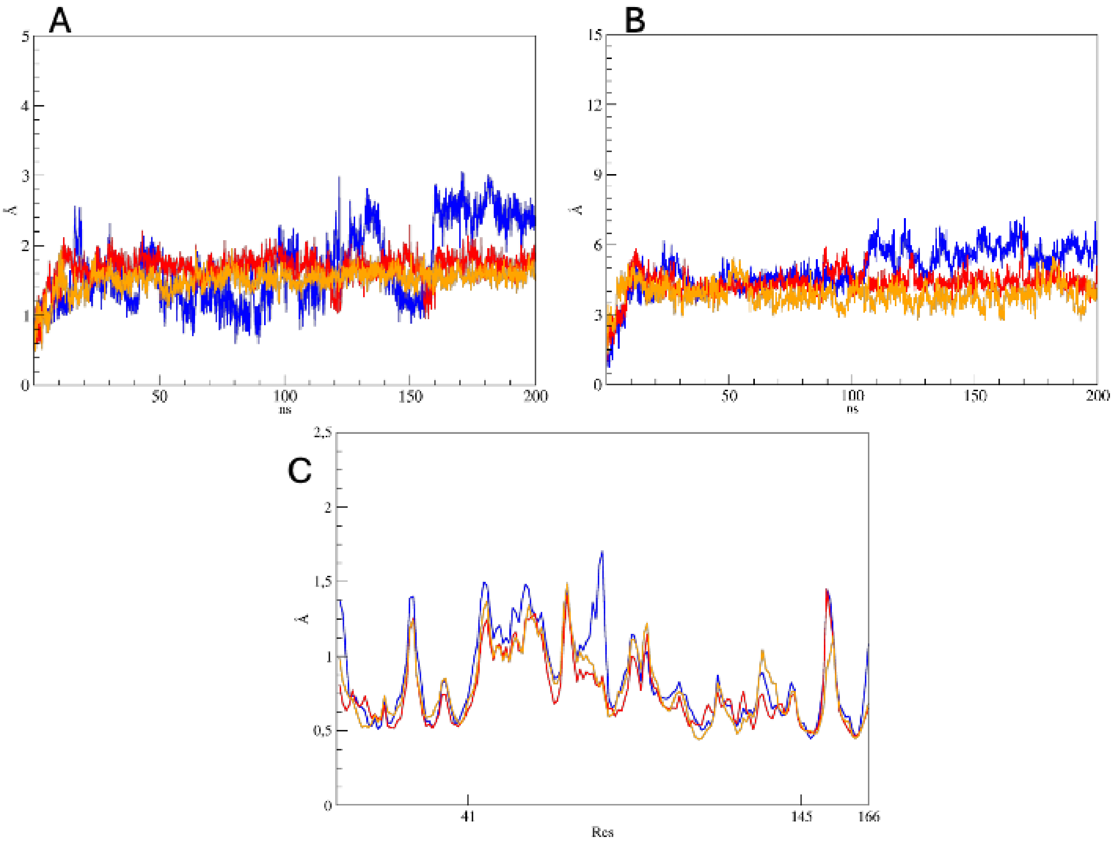


**Figure S4**. GOLD ChemPLP-derived 3CL$^{pro}$-PG401 complex during 200 ns of molecular dynamics simulations. In all panels, R1 is shown in blue, R2 in red, and R3 in orange. **(A)** RMSD of the 3CL$^{pro}$-PG401 complex. **(B)** RMSD of the 3CL$^{pro}$ binding-pocket residues. **(C)** RMSF profile of key binding-pocket residues, with residues involved in ligand interactions highlighted.

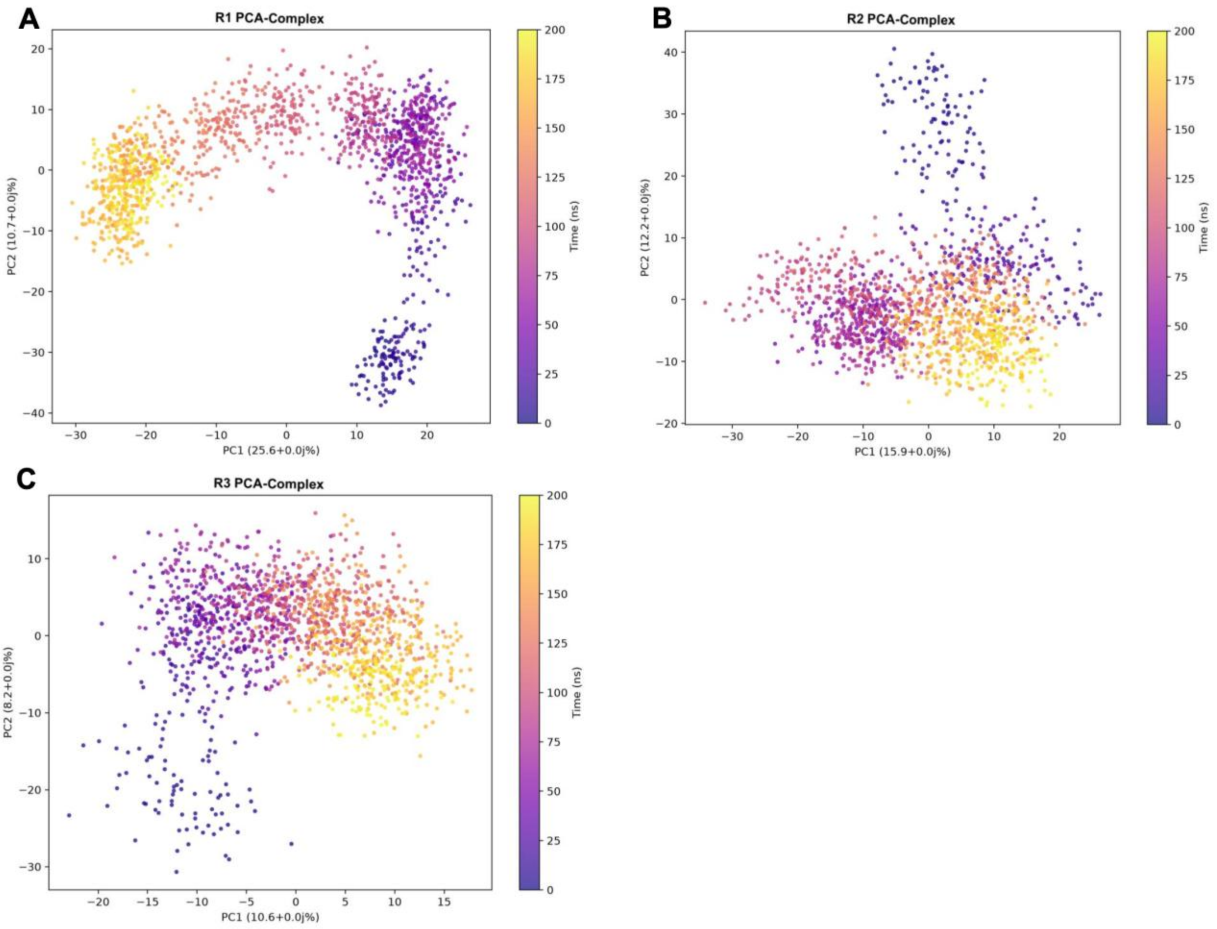


**Figure S5.** PCA of the 3CL$^{pro}$-PG401 complex obtained from the ChemPLP-generated binding pose. PCA projections along the PC1 and PC2 for the three independent replicas: (**A**) R1, (**B**) R2, and (**C**) R3. The color scale represents simulation time (0-200 ns), illustrating the conformational sampling of the complex throughout the trajectories.

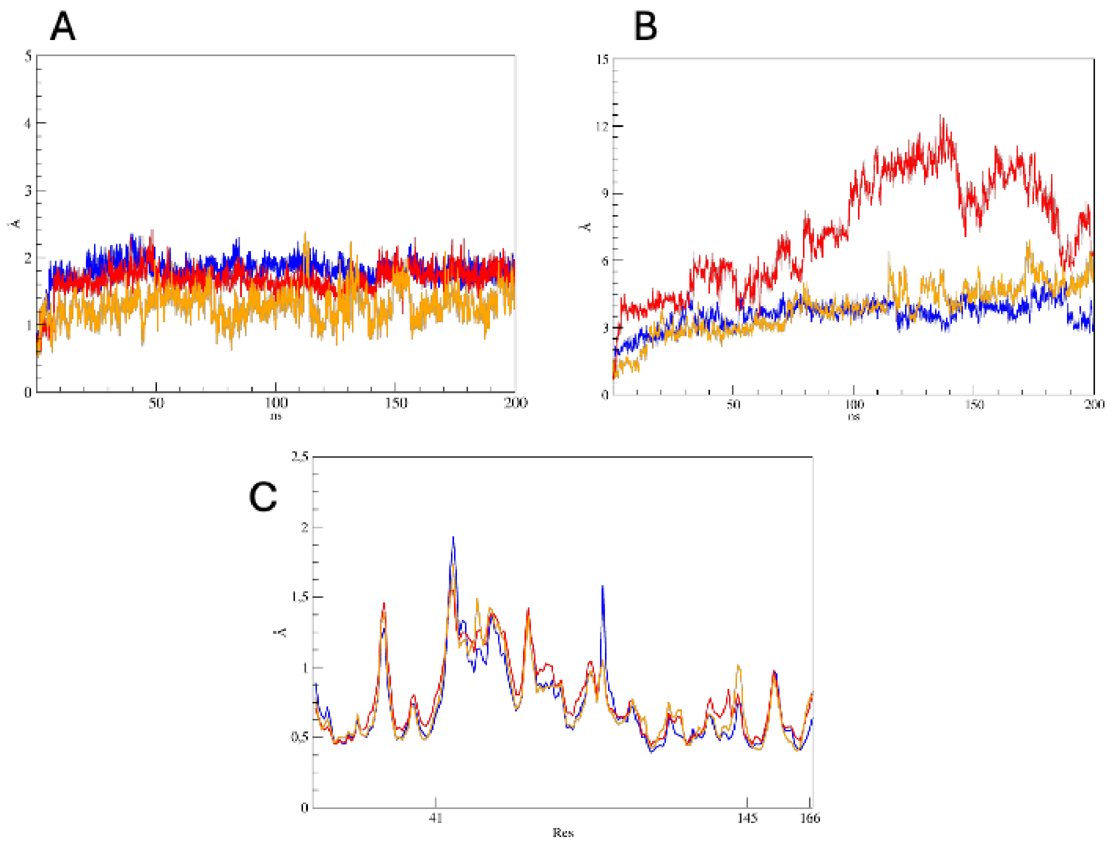


**Figure S6**. GOLD GoldScore-derived 3CL$^{pro}$-PG401 complex during 200 ns of molecular dynamics simulations. In all panels, R1 is shown in blue, R2 in red, and R3 in orange. **(A)** RMSD of the 3CL$^{pro}$-PG401 complex. **(B)** RMSD of the 3CL$^{pro}$ binding-pocket residues. **(C)** RMSF profile of key binding-pocket residues, with residues involved in ligand interactions highlighted.

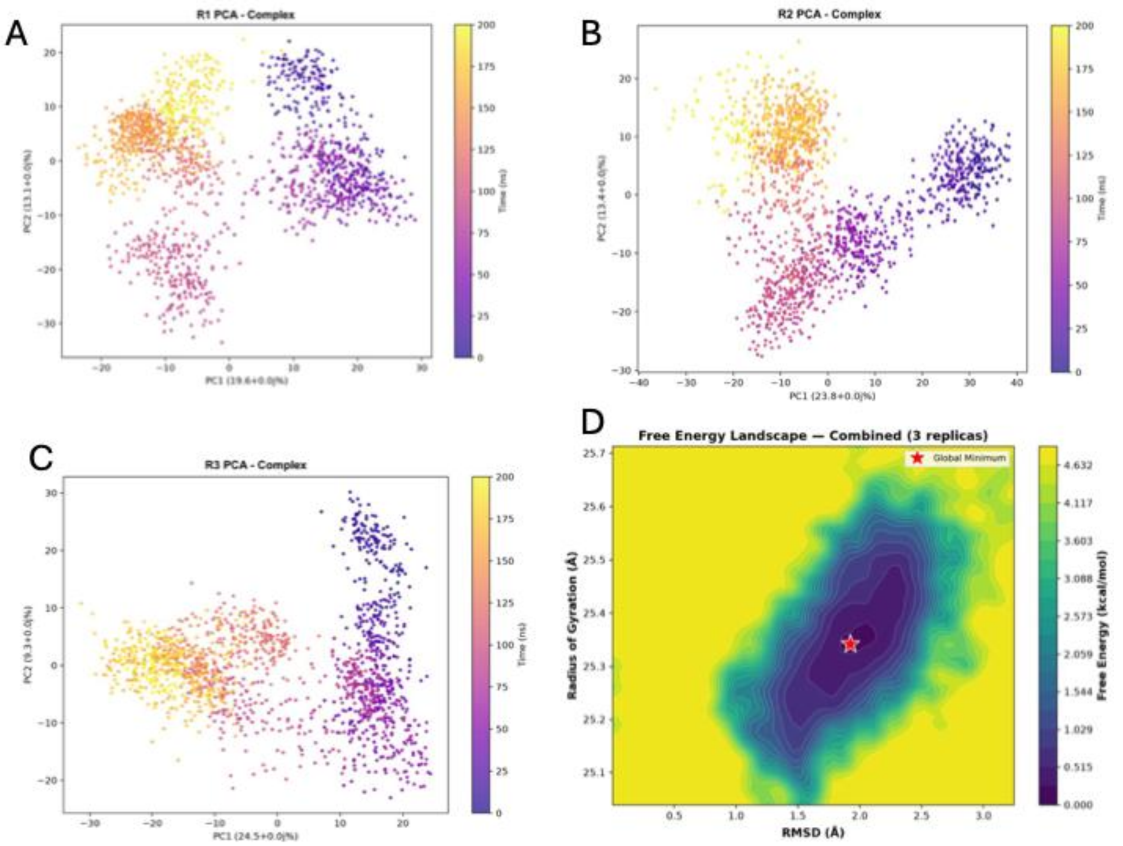


**Figure S7.** PCA of the 3CL$^{pro}$-PG401 complex obtained from the GoldScore-generated binding pose. PCA projections along the PC1 and PC2 for the three independent replicas: (**A**) R1, (**B**) R2, and (**C**) R3. The color scale represents simulation time (0-200 ns), illustrating the conformational sampling of the complex throughout the trajectories. (**D**) FEL of the 3CL$^{pro}$-PG401 complex projected onto RMSD and Rg, with the global minimum indicated by the red star.

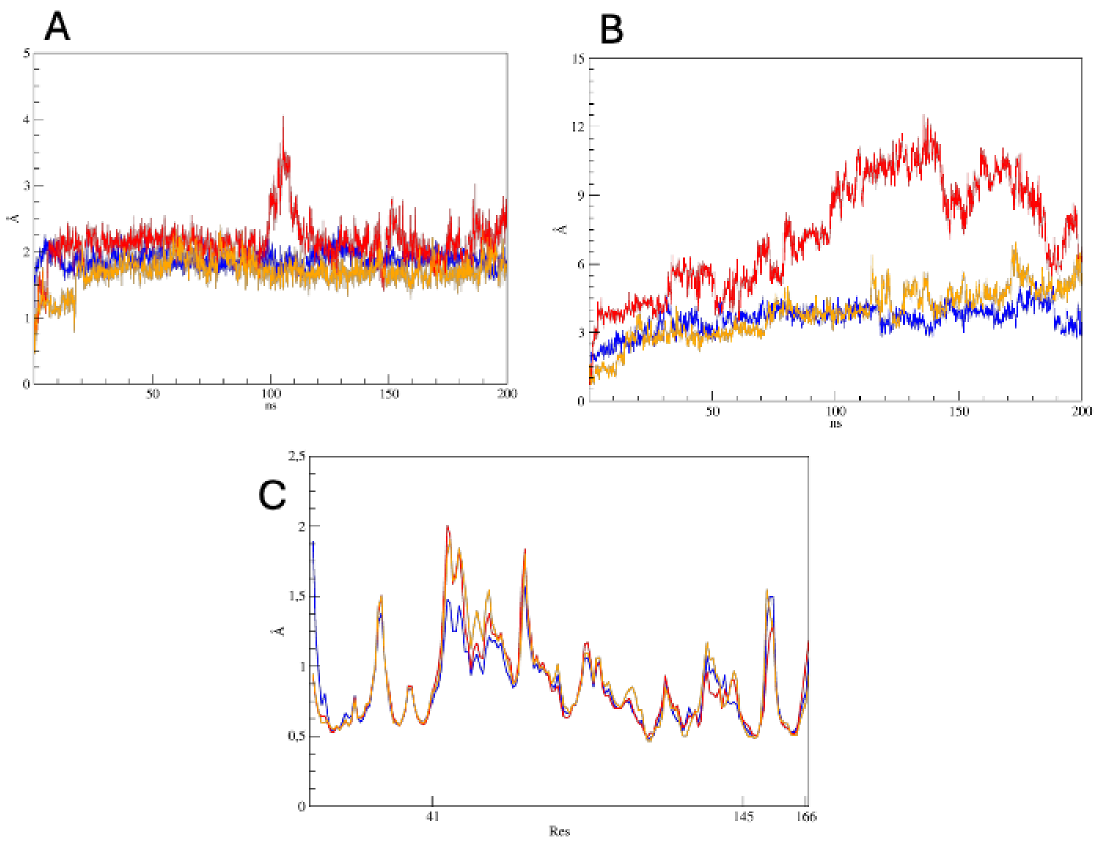


**Figure S8.** GOLD AutoDock Vina-derived 3CL$^{pro}$-PG401 complex during 200 ns of molecular dynamics simulations. In all panels, R1 is shown in blue, R2 in red, and R3 in orange. **(A)** RMSD of the 3CL$^{pro}$-PG401 complex. **(B)** RMSD of the 3CL$^{pro}$ binding-pocket residues. **(C)** RMSF profile of key binding-pocket residues, with residues involved in ligand interactions highlighted.

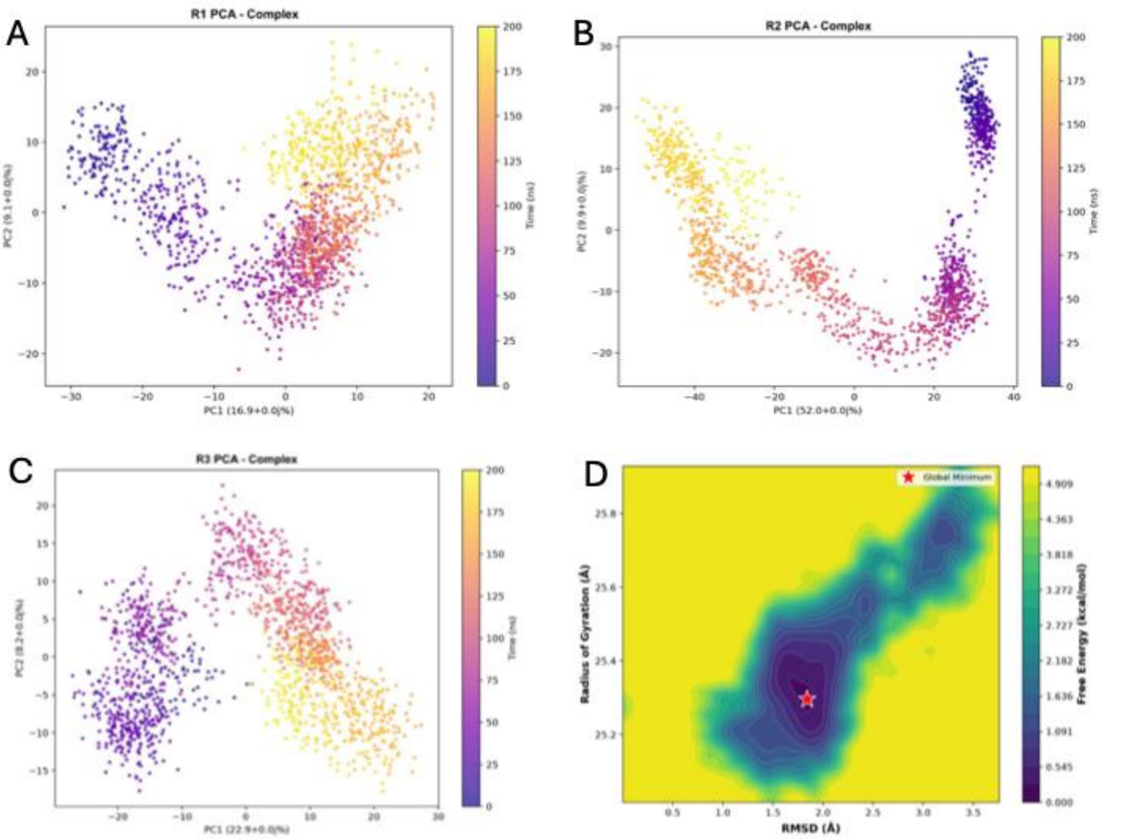


**Figure S9.** PCA of the $3CL^{pro}$-PG401 complex obtained from the AutoDock Vina-generated binding pose. PCA projections along the PC1 and PC2 for the three independent replicas: (**A**) R1, (**B**) R2, and (**C**) R3. The color scale represents simulation time (0-200 ns), illustrating the conformational sampling of the complex throughout the trajectories. (**D**) FEL of the $3CL^{pro}$-PG401 complex projected onto RMSD and Rg, with the global minimum indicated by the red star.

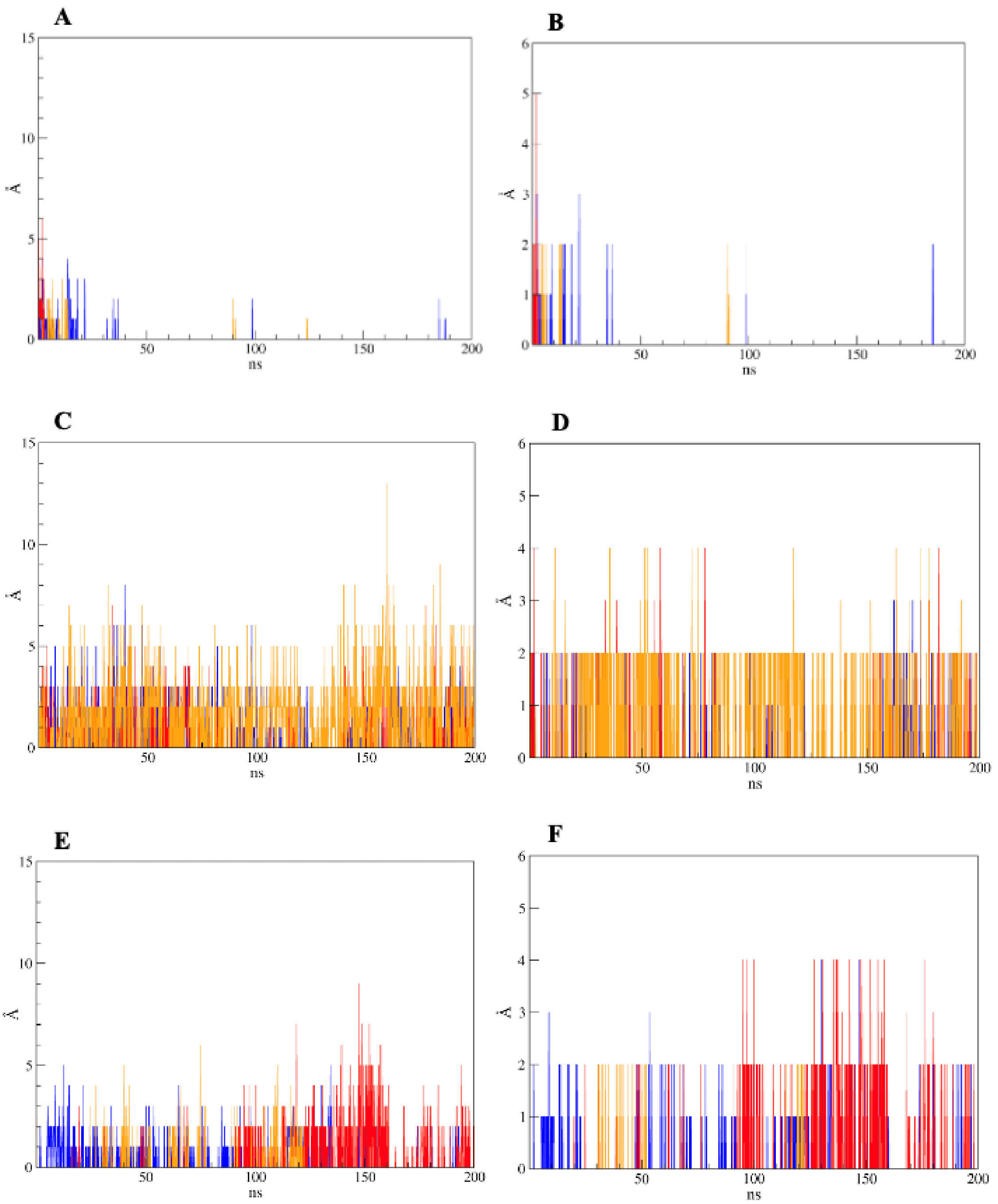


**Figure S10.** H-bond interactions between the $PL^{pro}$-PG 401 complex over 200 ns of molecular dynamics simulations. In all graphs R1 is colored in blue, R2 in red, and R3 in orange. **A** and **B** correspond to H-bonds predicted between BL2loop and only residue Y268, respectively, based on the pose obtained using the ChemPLP docking scoring function. S2. **C** and **D** correspond to H-bonds predicted between BL2loop and only residue Y268, respectively, obtained with the GoldScore function. S2. **E** and **F** correspond to H-bonds predicted between BL2loop and only residue Y268, respectively, derived from the AutoDock Vina. Each plot depicts the time-dependent number of hydrogen bonds between PG_401 and SARS-CoV-2 $PL^{pro}$.

**Table S8.** RMSF values in angstromns for $PL^{pro}$'s BL2loop residues. All three replicates of each scoring method.

| | GOLD - ChemPLP | | | GOLD - GoldScore | | | AutoDock Vina | | |
|---|---|---|---|---|---|---|---|---|---|
| Residue | R1 | R2 | R3 | R1 | R2 | R3 | R1 | R2 | R3 |
| 266 | 1.6684 | 2.1653 | 1.3006 | 1.4457 | 1.2738 | 1.0758 | 1.0757 | 1.0766 | 1.2602 |
| 267 | 3.1207 | 4.0507 | 2.3617 | 1.7729 | 1.5018 | 1.2761 | 1.3512 | 1.5513 | 2.6464 |
| 268 | 4.7871 | 5.4926 | 3.957 | 3.2628 | 1.662 | 1.2569 | 1.4768 | 2.5047 | 4.5992 |
| 269 | 4.1568 | 4.1557 | 3.4478 | 3.4686 | 1.6578 | 1.2495 | 1.4412 | 2.3688 | 4.6183 |
| 270 | 1.9731 | 1.8155 | 1.6762 | 1.5986 | 1.3281 | 1.2446 | 1.2857 | 1.4143 | 2.2518 |
| 271 | 0.9106 | 0.9297 | 0.9664 | 0.9838 | 1.0119 | 1.0278 | 1.0053 | 0.9379 | 1.0688 |

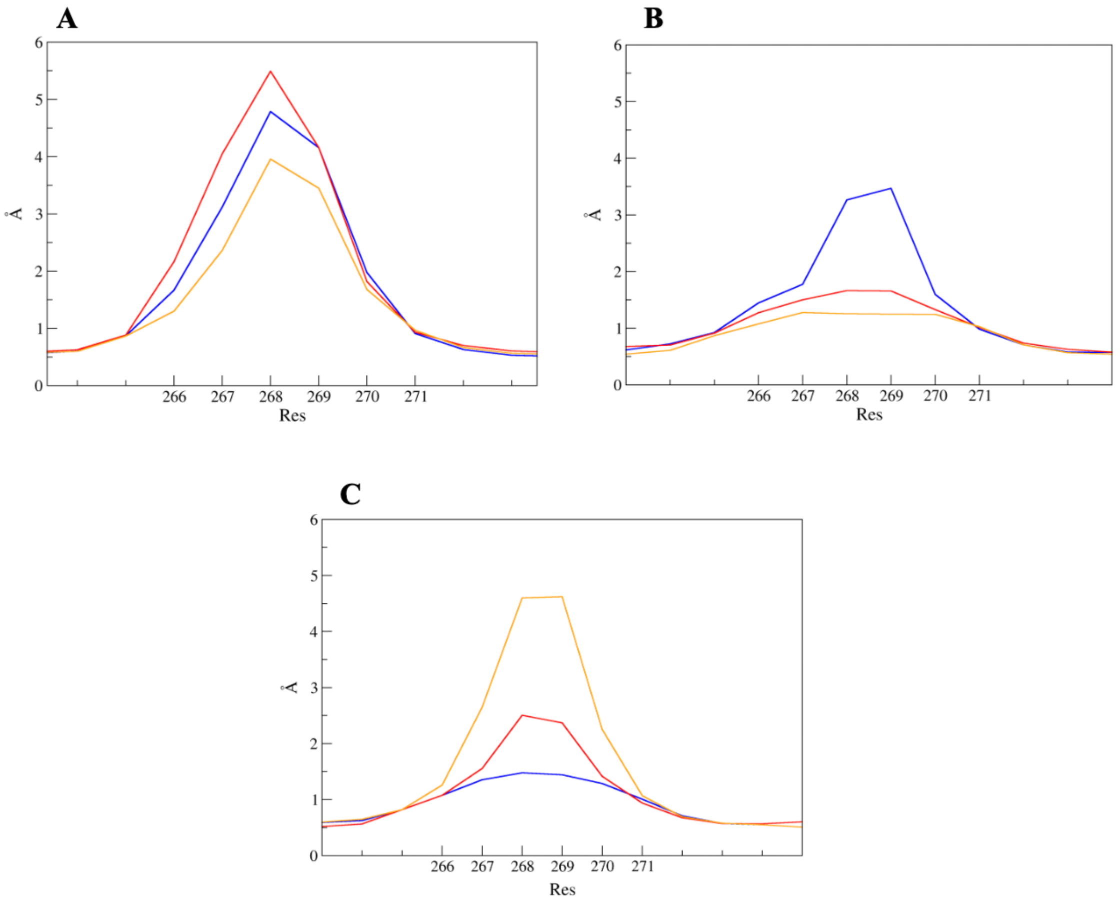


**Figure S11.** RMSF of the residues forming the BL2loop of $PL^{pro}$ over 200 ns of molecular dynamics simulations. In all graphs, R1 is shown in blue, R2 in red, and R3 in orange. (**A**) GOLD–ChemPLP $PL^{pro}$–PG_401 complex. (**B**) GOLD–GoldScore $PL^{pro}$–PG_401 complex. (**C**) AutoDock Vina $PL^{pro}$–PG_401 complex.
